# Enzymatic Supercoiling of Bacterial Chromosomes Facilitates Genome Manipulation

**DOI:** 10.1021/acssynbio.2c00353

**Published:** 2022-08-23

**Authors:** Hironobu Fujita, Ayane Osaku, Yuto Sakane, Koki Yoshida, Kayoko Yamada, Seia Nara, Takahito Mukai, Masayuki Su’etsugu

**Affiliations:** Department of Life Science, College of Science, Rikkyo University, Tokyo 171-8501, Japan

**Keywords:** chromosome topology, replication, electroporation, conjugation, genome splitting, genome swap

## Abstract

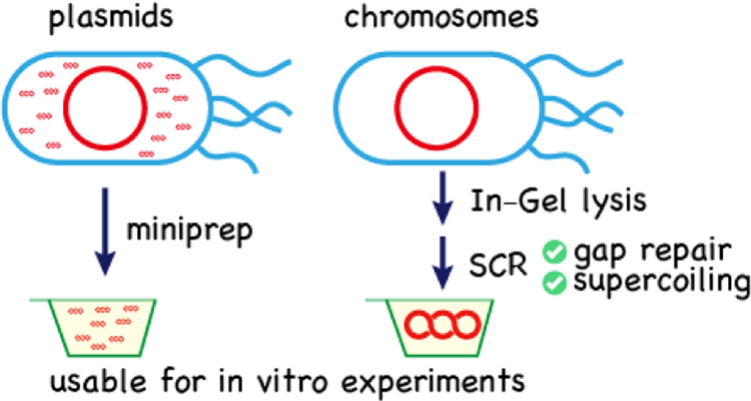

The physical stability of bacterial chromosomes is important for their in vitro manipulation, while genetic stability is important in vivo. However, extracted naked chromosomes in the open circular form are fragile due to nicks and gaps. Using a nick/gap repair and negative supercoiling reaction (named SCR), we first achieved the negative supercoiling of the whole genomes extracted from *Escherichia coli* and *Vibrio natriegens* cells. Supercoiled chromosomes of 0.2–4.6 megabase (Mb) were separated by size using a conventional agarose gel electrophoresis and served as DNA size markers. We also achieved the enzymatic replication of 1–2 Mb chromosomes using the reconstituted *E. coli* replication-cycle reaction (RCR). Electroporation-ready 1 Mb chromosomes were prepared by a modified SCR performed at a low salt concentration (L-SCR) and directly introduced into commercial electrocompetent *E. coli* cells. Since successful electroporation relies on the genetic stability of a chromosome in cells, genetically stable 1 Mb chromosomes were developed according to a portable chromosome format (PCF). Using physically and genetically stabilized chromosomes, the democratization of genome synthetic biology will be greatly accelerated.

In the era of genome synthetic biology, there is a need for technologies to routinely manipulate and analyze bacterial chromosomes in vitro.^1−4^ However, unlike small plasmids, bacterial chromosomes are fragile and difficult to handle when extracted from cells.^4^ In the most successful approach,^5−8^ a bacterial chromosome was cloned in yeast cells, extracted from the yeast cells in an agarose plug, and transplanted to *Mycoplasma capricolum* cells to boot up the engineered or heterologous genome. *M. capricolum* has an exceptionally high competency to uptake a chromosome as large as 1.2 megabase (Mb).^5,8^ Because *M. capricolum* cannot be converted to evolutionally distant bacteria,^8^ the phylogenetic range of competent host bacteria must be expanded. Recently, we succeeded in the transformation of *Escherichia coli* via electroporation with a 1.0 Mb chromosome purified using a bacterial artificial chromosome (BAC) purification kit.^9^ A population of negatively supercoiled 1 Mb chromosomes survived the purification and electroporation processes, probably owing to the compacted structure.^10^ Furthermore, these supercoiled chromosomes were separated by size using conventional agarose gel electrophoresis.^9^ These findings suggested that even larger chromosomes might be manipulatable when covalently closed and negatively supercoiled.

There are two traditional methods for estimating the sizes of bacterial chromosomes and megabase-sized plasmids using agarose gel electrophoresis: pulsed-field gel electrophoresis (PFGE)^11,12^ and modified Eckhardt gel electrophoresis.^13−16^ In PFGE, small agarose plugs containing naked chromosomes are embedded in a large agarose gel as samples for electrophoresis. Double-stranded DNA (dsDNA) molecules physically or enzymatically linearized are resolved by size, while large circular dsDNA molecules in the open circular form are trapped near the start.^6,17,18^ In successful cases, circular dsDNA molecules, probably in the supercoiled form, appear in the gel.^19^ However, there is no established way of estimating the size of supercoiled megabase-sized dsDNA molecules by PFGE.^20^ On the other hand, the modified Eckhardt method employs a normal agarose gel electrophoresis and resolves supercoiled dsDNA molecules. Bacterial cells are gently lysed in wells and directly used as the electrophoresis samples. Intact supercoiled chromosomes and plasmids are run on a 0.8–0.9% agarose gel for a half day to a few days. Up to 2.1 Mb plasmids can be detected and separated by size.^15,16^ The supercoiled genomic chromosome might appear in the gel only in a successful case.^16^ Although the two agarose gel electrophoresis methods are authentic,^21^ it is ideal if supercoiled chromosomes could be prepared in a PCR tube, directly applied to wells, and separated by size using a conventional agarose gel electrophoresis method within a few hours at room temperature.

Establishment of genome-reduced strains and genome-split strains of *E. coli* facilitates whole genome engineering.^9,22−24^ In nature, a considerable portion of bacteria, but not *E. coli*, have a multipartite genome.^25^ For example, most *Vibrio* species have two chromosomes (Chr1 and Chr2).^19,26,27^ Since *Vibrio* is closely related to *E. coli*,^28^ the chromosome replication origin and partitioning system of *Vibrio* Chr2 has been used to develop a secondary chromosome in *E. coli*.^29−31^ The partitioning system of the *E. coli* F plasmid (*sopABC*) has also been employed to maintain a sub-megabase-sized chromosome in *E. coli*.^22,31−33^ By combining all of these efforts, we recently established a tripartite-genome strain RGF138^9^ from a genome-reduced (4.65 → 2.98 Mb) *E. coli* strain DGF-298W.^24^ RGF138 looked normally and grew twice slowly as the parent. Among the three split-chromosomes (1.12, 0.84, and 1.02 Mb), the second and third ones were individually introduced via electroporation into an *E. coli* cloning strain HST08 as an extra chromosome.^9^ This meant that the two chromosomes are “portable.” In contrast, the first one was not portable, probably due to the burden of the duplication of the genomic core region or due to the lack of any additional chromosome partitioning system and segregation system.^9^ In this study, we show that enzymatically supercoiled bacterial chromosomes can be handled in vitro and are useful for genome analysis and chromosome implantation. Also, we show that the genetic stability of chromosomes can be improved according to a portable chromosome format.

## Results and Discussion

### Enzymatic Supercoiling of Bacterial Chromosomes

**Figure 1:**
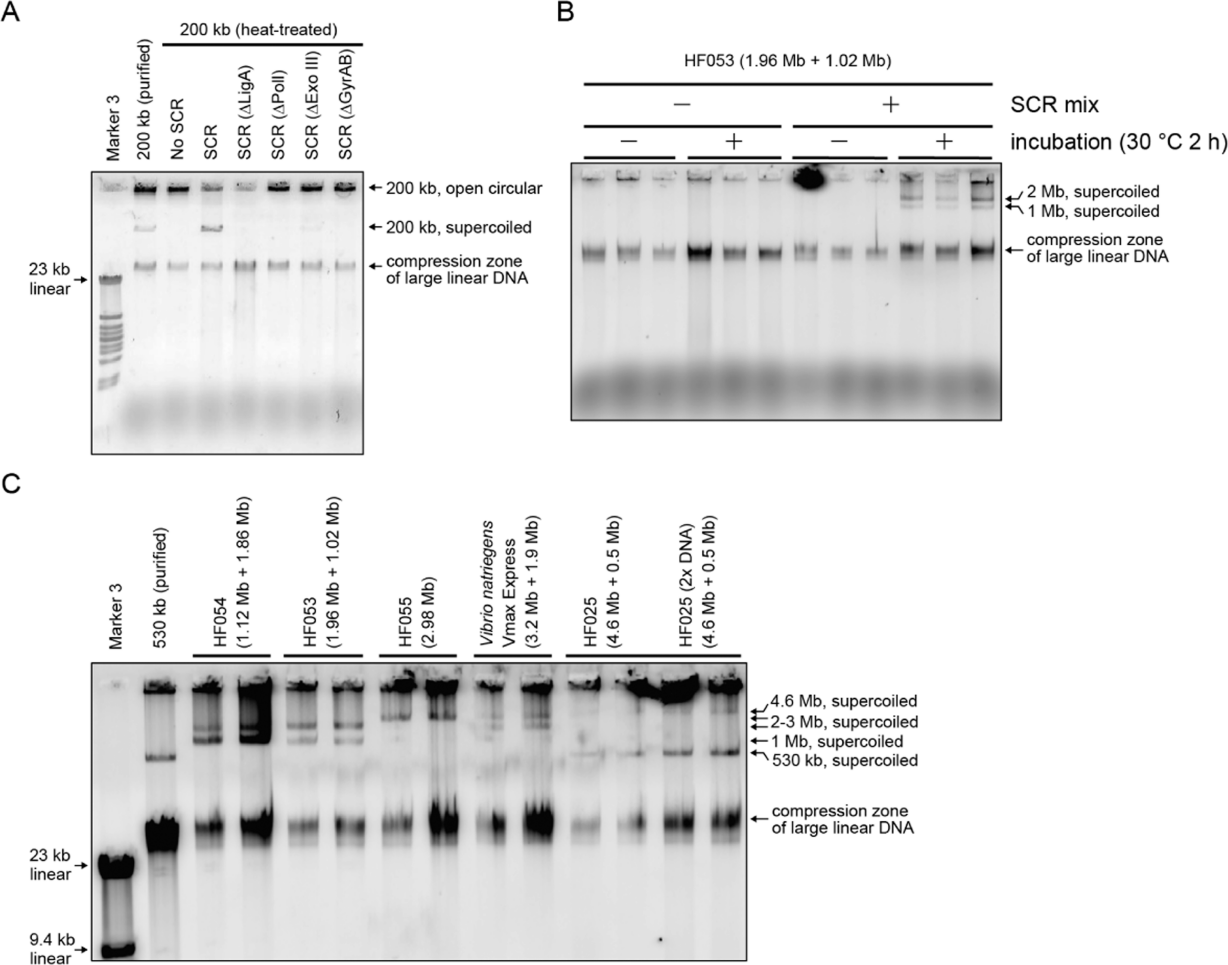
Supercoiling and repair reaction (SCR) supercoils open circular forms of bacterial chromosomes. (A) Agarose gel electrophoresis analysis (0.5% gel) of 200 kb plasmids incubated with indicated enzymes. The 200 kb plasmid had been heat-treated to introduce nicks (70 °C for 50 min). The open circular plasmids were trapped in the wells, while the supercoiled plasmids migrated into the gel. All four enzymes were essential or important for SCR. (B) Agarose gel electrophoresis analysis (0.3% SeaKem Gold gel, 0.5 × Tris borate-EDTA (TBE), 40 V 60 min) of the SCR products of the two chromosomes of *E. coli* HF053 cells extracted in an agarose plug. Because megabase-sized chromosome samples as well as their SCR products are often highly viscous, a few replicates of each sample were applied and analyzed throughout this study. (C) Agarose gel electrophoresis analysis (0.3% SeaKem Gold gel, 0.5 × TBE, 40 V 90 min) of the SCR products of bacterial chromosomes ranging from 1.0 to 4.6 Mb in size.

Plasmid’s topology can be converted from open circular to highly supercoiled using *E. coli* enzyme mixtures composed of DNA polymerase I (*Pol*I), DNA ligase (LigA), Exonuclease III (*Exo*III), and DNA gyrase (GyrAB). A plasmid supercoiling reagent was commercially available from a company. The nicks and gaps on open circular plasmids are filled and fused by *Pol*I and ligase,^34^ while *Exo*III makes easy-to-repair gaps.^35,36^ Gyrase helps in the supercoiling of scarless plasmids in an ATP-dependent manner.^37,38^ Since these reactions are involved in the chromosome replication cycle of *E. coli*, these enzymes are included in the reconstituted *E. coli* replication-cycle reaction (RCR).^31,39^ Because RCR produced supercoiled plasmids of up to 1.1 Mb in size in a PCR tube,^31^ we assumed that enzymatic supercoiling of open circular bacterial chromosomes could be mediated by a subset of RCR enzymes and buffers. This dedicated reaction was named “SuperCoiling and Repair Reaction” (SCR). SCR reagents were established using a 200 kb plasmid as a model DNA template. By heat treatment which introduces nicks, supercoiled plasmids were converted to be open circular. As expected, a significant proportion of the open circular plasmids were supercoiled using a complete reaction mixture with the four enzymes in the RCR buffer (Figure 1A). Remember that using the conventional agarose gel electrophoresis, open circular large plasmids are trapped in the wells, and large linear dsDNAs migrate to the compressed zone, while the supercoiled large plasmids are separated by size.^39,40^ Omission of any of *Pol*I, LigA, and GyrAB fully impaired the reaction, while *Exo*III contributed to the maximum reaction efficiency (Figure 1A), probably by eliminating damaged 3′-OH ends^35^ and apurinic/apyrimidinic sites.^36^ To evaluate the maximum potential of SCR, larger bacterial chromosomes were prepared using the conventional agarose plug method from several *E. coli* strains and *Vibrio natriegens* Vmax Express (Codex DNA, Inc.). Without SCR, supercoiled chromosomes were hardly detectable using conventional agarose gel electrophoresis (Figure 1B), which is consistent with the recent findings that the genomic chromosome in *E. coli* is often nicked and gapped.^41,42^ Furthermore, chromosomes may be damaged during the gel extraction step using β-agarase at 65 °C for 10 min. In contrast, the SCR products of bacterial chromosomes ranging from 1.0 to 4.6 Mb in size were detected and separated by size in a 0.3% SeaKem Gold Agarose gel^43^ by the conventional agarose gel electrophoresis performed for 1–1.5 h at room temperature (Figure 1B,C). The overnight SCR protocol was 24 °C for >10 h (up to 4–5 Mb), while the quick protocol was 30 °C for 30 min (for 200 kb), 2 h (for 3 Mb), and 3 h (for 4–5 Mb), depending on the total length of chromosomes (Table 1). Thus, even the wild-type genomic chromosomes of *E. coli* (4.6 Mb) and *V. natriegens* (3.2 Mb + 1.9 Mb) were supercoiled and manipulated for the subsequent analyses. In summary, we noticed that open circular bacterial chromosomes can be supercoiled in vitro in a similar fashion as for small plasmids.

**Table 1:** SCR/L-SCR and RCR Protocols

SCR/L-SCR: 37 °C for a few hours or 24 °C overnight	
10× SCR/L-SCR buffer 1	1 μL
10× SCR/L-SCR buffer 2	1 μL
10× SCR/L-SCR enzyme mix	1 μL
template DNA	1 μL (up to 2 μL)
Milli-Q water	6 μL (or 5 μL)
total volume in the PCR tube	10 μL
RCR: 20 cycles at 37 °C for 30 s and 22 °C for 60 min	
10× RCR buffer 1	1 μL
10× RCR buffer 2	1 μL
10× RCR enzyme mix	0.5 μL
20× RecGJ *Exo*III	0.25 μL
60 ng/μL λ phage DNA	0.5 μL
2% DMSO	0.5 μL
40% dextran	0.625 μL
template DNA	1 μL
Milli-Q water	4.625 μL
total volume in the PCR tube	10 μL

### Size Separation of Supercoiled Bacterial Chromosomes

**Figure 2:**
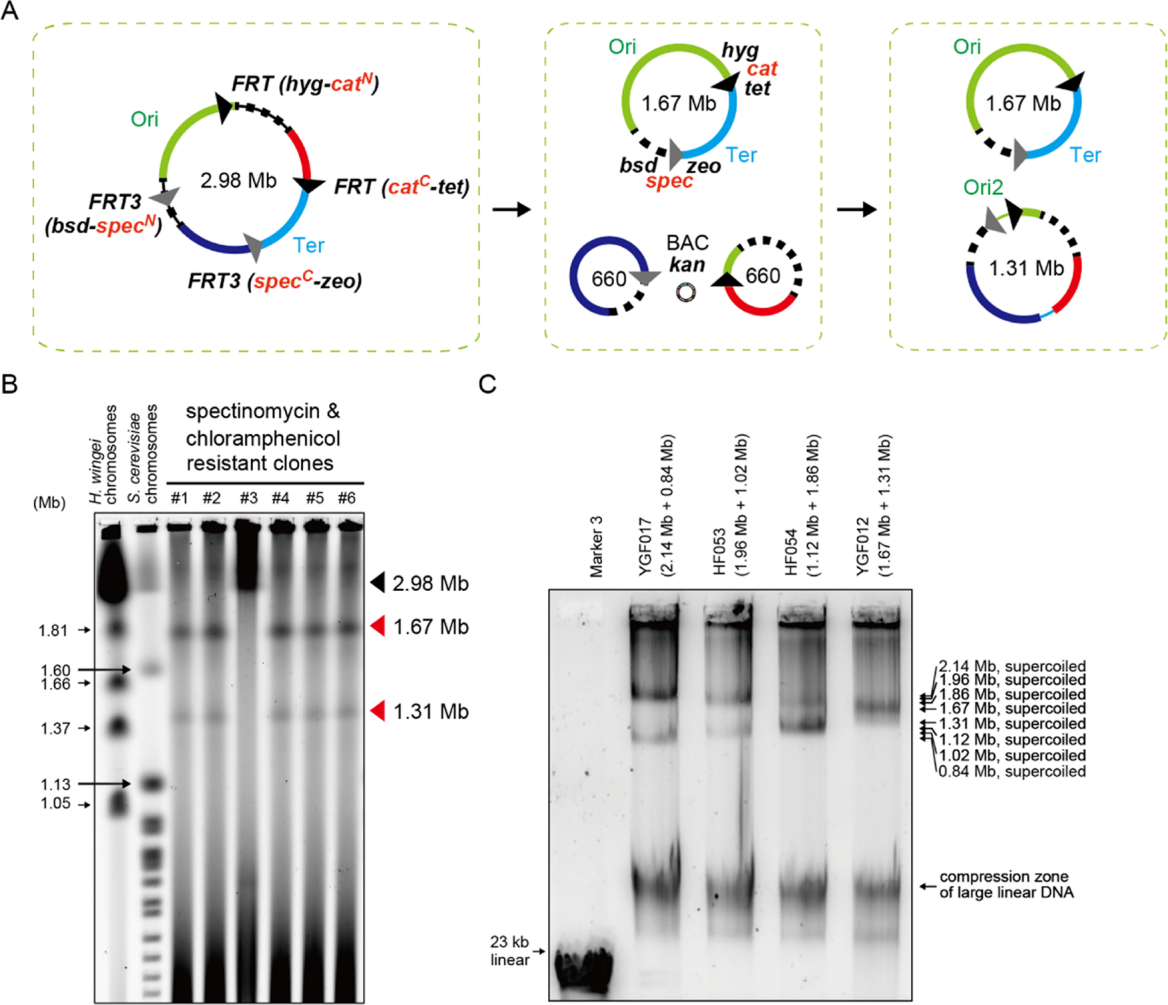
Evaluating the resolution of the conventional agarose gel electrophoresis method for supercoiled circular chromosomes within the range of about 1–2 Mb. (A) Scheme for the dual Flp-POP cloning method to develop a bipartite-genome *E. coli* strain YGF012. Two genomic regions were marked by two *FRT* sequences and two *FRT3* sequences via λ-red recombination, together with four kinds of the indicated antibiotic marker genes. Abbreviations: *hyg* for hygromycin, *tet* for tetracycline, *bsd* for Blasticidin S, *zeo* for Zeocin, *cat* for chloramphenicol, *spec* for spectinomycin, and *kan* for kanamycin. Upon induction of the flippase, the two *FRT/FRT3*-flanked regions were popped-out to develop two circular DNA molecules, while the main chromosome gained the full-length *cat* and *spec* marker genes carrying an *FRT/FRT3* sequence inside their open reading frames which had been separated by *FRT/FRT3* sequences. Upon induction of the HK022 phage integrase, the two circular DNAs were integrated into the two *attB*^HK022^ sites in the BAC vector via the *attB-attP* recombination to develop a new 1.31 Mb chromosome. (B) PFGE analysis (1% PrimeGel Agarose GOLD 3-40K, 0.5 × TBE, 24 h, 6 V/cm, 120°, 60–120 s switch time ramp) of the chromosomes of six colonies showing resistance to spectinomycin and chloramphenicol. Five clones have the bipartite-genome configuration, while one has a single chromosome. Clone #5 was renamed YGF012. The marker chromosomes from yeast cells did not migrate in a fully size-dependent manner. (C) Agarose gel electrophoresis analysis (0.3% SeaKem Gold gel, 0.5 × TBE, 40 V 120 min) of the SCR products of bacterial chromosomes ranging from 0.84 to 2.14 Mb in size.

To evaluate the resolution of the conventional agarose gel electrophoresis using 0.3% gels, we used *E. coli* chromosomes of various sizes: 0.84, 1.02, 1.12, 1.31, 1.67, 1.86, 1.96, and 2.14 Mb. These chromosomes were prepared from several bipartite-genome strains of *E. coli*. The pairs of 1.02 Mb + 1.96 Mb and 1.12 Mb + 1.86 Mb were from HF053 and HF054, respectively,^9^ while the pairs of 0.84 Mb + 2.14 Mb and 1.31 Mb + 1.67 Mb were from newly developed bipartite-genome strains YGF017 and YGF012, respectively. The two chromosomes of YGF017 and YGF012 were constructed by the dual Flp-POP cloning method (Figure 2A), a modified Flp-POP cloning method^31^ developed for cloning two genomic regions into a single BAC vector. Upon a Flp-POP cloning, a genomic region flanked by *FRT* sites is flipped out by flippase as an origin-less circular DNA and integrated into a BAC vector by HK022 phage integrase.^31^ This Flp-*FRT* recombination combines the N-terminal and C-terminal halves of an antibiotic selection marker gene intervened by the *FRT* sites.^31^ The 1.31 and 1.67 Mb chromosomes were confirmed by PFGE analysis (Figure 2B), while the 0.84 and 2.14 Mb chromosomes were confirmed using SCR (Figure S1A). These eight split-chromosomes that ran for 2 h on a 0.3% agarose gel were separated by size (Figures 2C and S1B). Two chromosomes of very similar sizes may not be resolved in the same lane. We believe that the conventional agarose gel electrophoresis provides acceptable resolution for supercoiled circular chromosomes in the range of about 1–2 Mb. The dual Flp-POP cloning method is useful for constructing large chromosomes of any size. The combination of SCR and the conventional agarose gel electrophoresis may greatly facilitate experiments using megabase-sized circular chromosomes.

### Enzymatic Replication of Bacterial Chromosomes

RCR is another way to produce supercoiled chromosomes. Our previous study suggests that RCR has the potential to replicate *oriC*-containing chromosomes of up to 1.1 Mb in size.^31^ Although the agarose gel electrophoresis analysis is a direct way of detecting supercoiled molecules produced by RCR, it was difficult to properly estimate the size and yield of these molecules. Using SCR products as supercoiled DNA size marker, we examined the maximum potential of RCR. RCR is composed of about 26 kinds of recombinant proteins and amplifies *oriC*-containing plasmids/chromosomes in an exponential manner.^39^ One of the main differences between this reconstituted system and the native cellular system is the lack of the replication restart mechanism from the site where a replisome dropped off.^44^ Another difference is the lack of mismatch repair systems. Thus, the processivity of the replisome limits the maximum size of RCR-amplifiable chromosomes. To prepare 1.12 and 1.96 Mb chromosomes containing an *oriC* locus, the chromosomes of bipartite-genome *E. coli* strains HF054 (1.12 Mb + 1.86 Mb) and HF053 (1.96 Mb + 1.02 Mb) were extracted in agarose plugs. Since RCR includes the SCR enzymes, nonreplicated but repaired and supercoiled products should be discriminated from RCR-replicated chromosomes. In principle, SCR products of *E. coli* cell-derived chromosomes are methylated and enzymatically digestible with *Dpn*I, while RCR-replicated chromosomes are unmethylated and thus resistant to *Dpn*I. This is the same as PCR products amplified from *E. coli* plasmids.^45^

**Figure 3:**
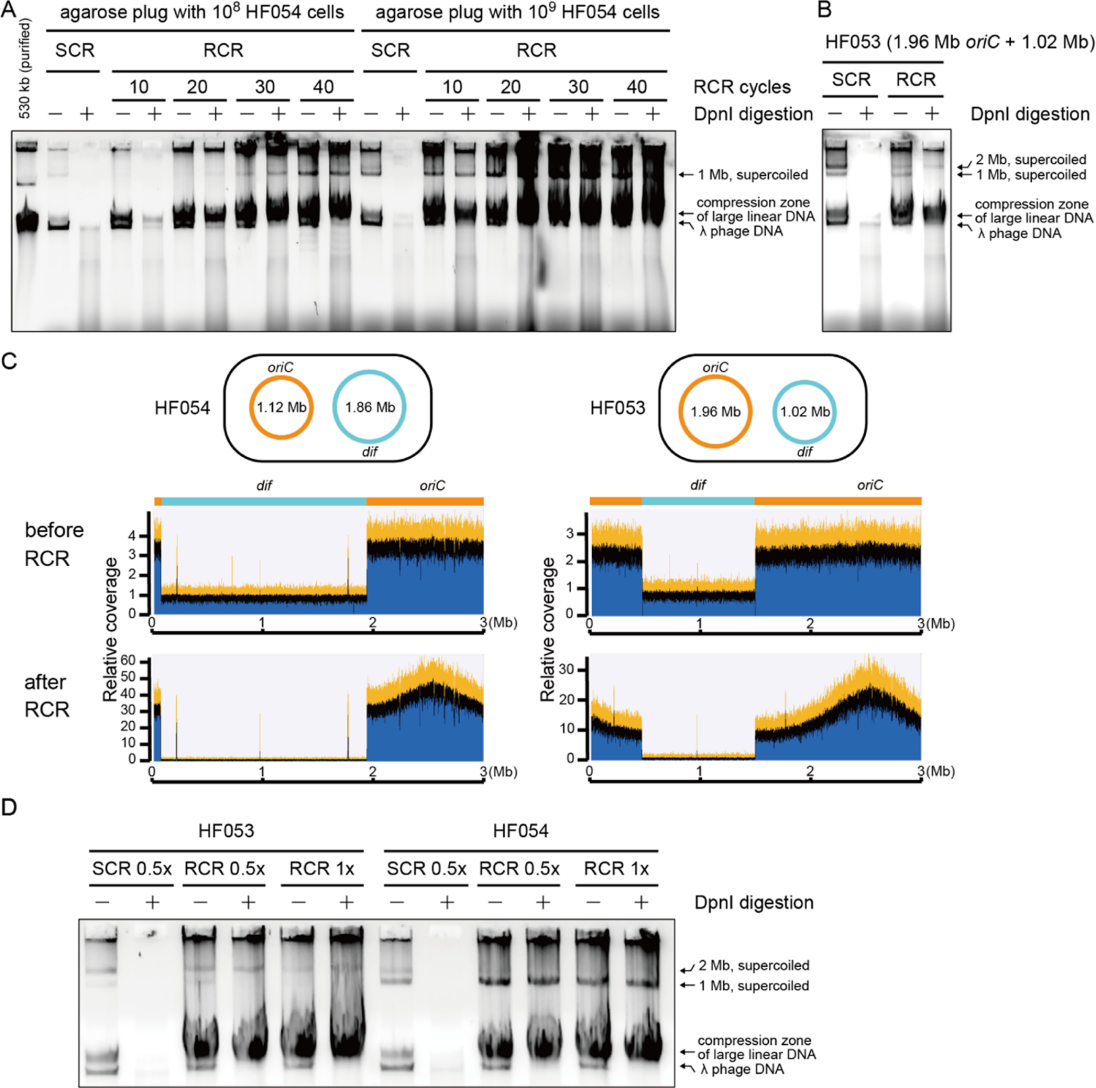
Evaluating the maximum size of bacterial chromosomes replicable by replication-cycle reaction (RCR). (A) Agarose gel electrophoresis analysis (0.3% SeaKem Gold gel, 0.5 × TBE, 40 V 60 min) of the SCR and RCR products of the HF054 chromosomes. The SCR and RCR products were incubated or not incubated with *Dpn*I prior to the electrophoresis. The number of RCR thermal cycle steps was indicated. (B) Agarose gel electrophoresis analysis of the SCR and RCR products of the HF053 chromosomes. The condition was fixed to an agarose plug with 10^9^ cells and 10 RCR thermal cycle steps. (C) NGS analyses of extracted chromosomes before and after RCR with the alignments to the HF033 reference genome (single chromosome). The relative average read depth of the *oriC*-containing chromosome compared to the *oriC*-less chromosome increased by about 9 and 4 times for HF054 and HF053, respectively. As for the after-RCR samples, many short reads were aligned and heaped on/around the *oriC* locus on the reference genome, indicating frequent failures during chromosome replication from *oriC*. (D) Agarose gel electrophoresis analysis of the SCR and RCR products of the HF053 and HF054 chromosomes. The usual amount of RCR and half the amounts of RCR and SCR enzymes were used, as indicated. The RCR thermal cycle steps were 20.

First, we performed a preliminary experiment to find an RCR protocol for the 1.12 Mb chromosome. Agarose plugs were made using either 10^8^ cells or 10^9^ cells. RCR reaction mixtures were incubated for 10–40 cycles at 37 °C for 30 s (for *oriC* firing) and 22 °C for 60 min (for extension). The SCR product from a 10^9^ plug yielded a clear band for 1.12 Mb, while the 1.12 Mb band from a 10^8^ plug was faint (Figure 3A). As expected, *Dpn*I-digested SCR products lack bands for any large DNA (Figure 3A). When using a 10^8^ plug, 1.12 Mb bands were visible on the gel after >20 cycles of RCR amplification and became clear after 40 cycles. These supercoiled 1.12 Mb chromosomes remained after *Dpn*I treatment (Figure 3A), indicating that this *oriC*-containing chromosome was replicated by RCR. Using a 10^9^ plug, clear bands for *Dpn*I-resistant chromosomes were observed even after 10 cycles (Figure 3A). Longer incubation rather produced a bunch of byproducts which made the reaction solution highly viscous. Then, the 1.96 Mb chromosome was amplified by RCR using this protocol (10^9^ plug and 10 cycles) and was shown as resistant to *Dpn*I (Figure 3B). Next-generation sequencing (NGS) analysis of chromosomes before and after RCR was performed. The RCR-amplification rates of the 1.12 and 1.96 Mb circular chromosomes were estimated as 9 times and 4 times, respectively, by roughly comparing the read depths (Figure 3C). We also observed a heap of reads around the *oriC* locus, indicating that the linear RCR byproducts were caused by the spontaneous drop-off of the replisomes and/or double-strand break during the reaction.

We then examined RCR mixtures with half the amount of enzymes. We assumed that the usual amount of enzymes was excess for a small number of megabase-sized chromosome molecules as the original RCR mixture was developed for the exponential amplification of small plasmids. After 20 cycles of RCR amplification, clear bands of *Dpn*I-resistant 1.12 and 1.96 Mb supercoiled chromosomes were detected (Figure 3D). Thus, we employ the half enzyme RCR protocol for >1 Mb chromosomes (Table 1). It is also indicated that the processivity of the reconstituted *E. coli* replisome in the bidirectional replication mode is up to 1.0 Mb. RCR replication of a 3 Mb chromosome has not yet been achieved in a reproducible manner. We think RCR is potentially very useful if the replisome processivity and the replication fidelity (reportedly ∼1.2 × 10^–8^ per base per replication cycle)^39^ would be greatly improved. On one hand, SCR is very handy and *oriC*-independent.

### Electroporation-Ready Chromosomes Prepared by SCR

**Figure 4:**
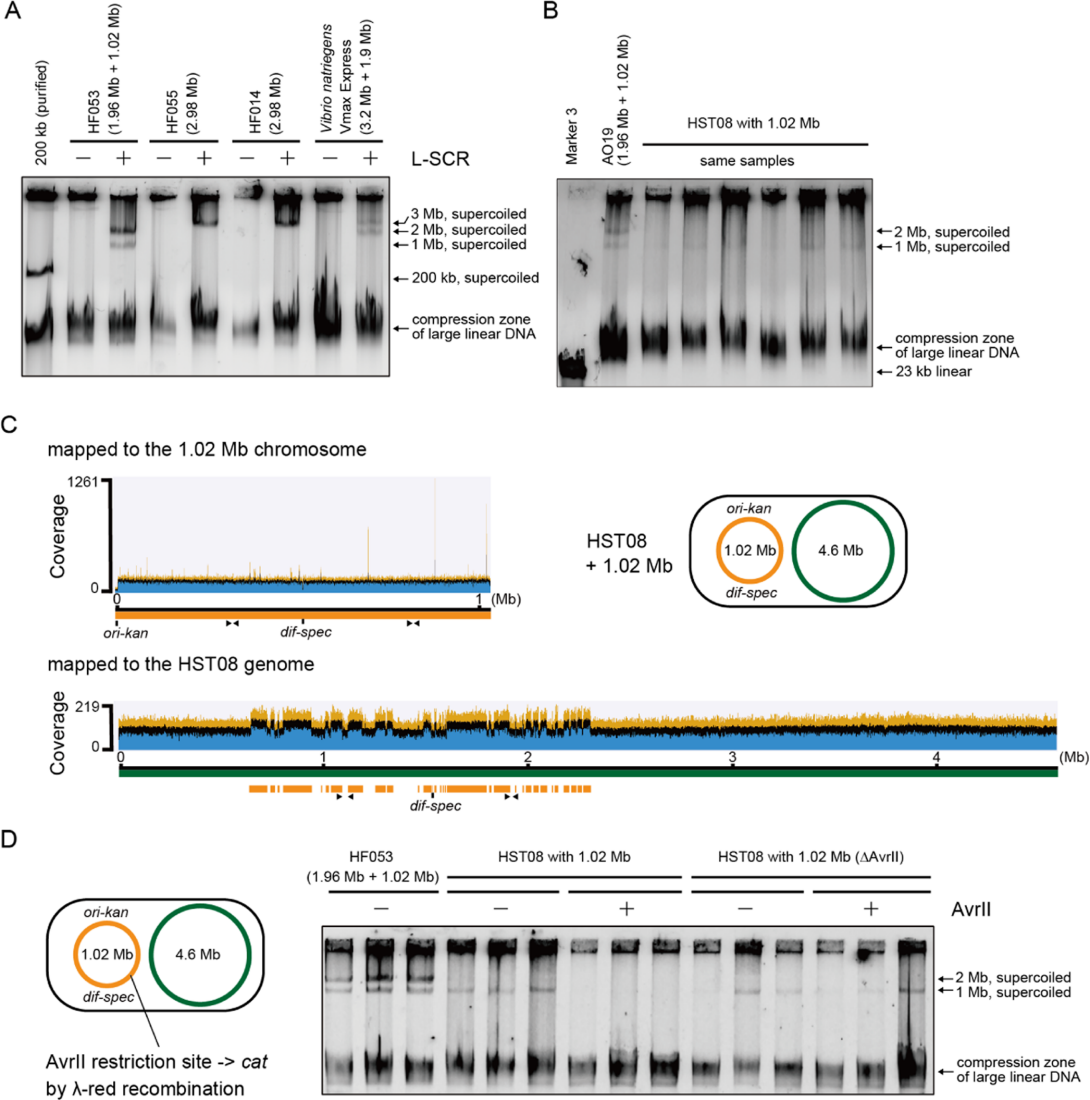
Electroporation-ready chromosomes can be prepared by low salt concentration SCR (L-SCR). (A) Agarose gel electrophoresis analysis (0.3% SeaKem Gold gel, 0.5 × TBE, 40 V 90 min) of the L-SCR products of bacterial chromosomes ranging from 1.02 to 3.2 Mb. (B) L-SCR products of the 1.02 Mb chromosome of AO19 were introduced into *E. coli* HST08 cells via electroporation. One hit colony (one of the three neighboring colonies) was analyzed by L-SCR. Agarose gel electrophoresis analysis (0.3% SeaKem Gold gel, 0.5 × TBE, 40 V 70 min) of the L-SCR products from AO19 and the transformed HST08 clone was performed. (C) NGS analysis of the HST08 clone carrying the 1.02 Mb chromosome for confirming the full-length 1.02 Mb chromosome in HST08. Mapping to the HST08 reference genome showed discontinuous heaps of the duplicated regions (in total 1.02 Mb). The *ori-kan* and *dif-spec* loci and the two junctions (indicated with arrows) used for colony PCR are shown on the maps. (D) Genetic modification of the 1.02 Mb chromosome in HST08 to remove the *Avr*II restriction site and the SCR incubation of the original and modified 1.02 Mb chromosome with or without *Avr*II (0.1 U/μL). Agarose gel electrophoresis analysis (0.3% SeaKem Gold gel, 0.5 × TBE, 40 V 60 min) of the SCR products was performed to examine their *Avr*II sensitivity and resistance.

A supercoiled 1 Mb chromosome can be implanted into *E. coli* via electroporation if the chromosome is genetically stable inside the host cells.^9^ In contrast, stretched 1 Mb DNA molecules are not only fragile but also even longer than a bacterial cell. Although purified and desalted DNA samples are usually used for electroporation, it is ideal if SCR products could be directly used for electroporation to avoid any physical damages of purification. We modified the buffer composition of SCR and found potassium acetate (KOAc) and dithiothreitol (DTT) unnecessary in the SCR buffer. Thus, we omitted KOAc and DTT. The final salt concentration was reduced from about 200 mM to about <50 mM (10 mM Mg(OAc)_2_, 20 mM Tris–HCl, 10 mM ammonium sulfate, and minor ingredients). This Low Salt Concentration SCR was named L-SCR (Table 1). L-SCR successfully produced supercoiled 1, 2, and 3 Mb chromosomes (Figure 4A). To examine the transferability of L-SCR products via electroporation, we prepared a 1.02 Mb chromosome carrying a *kan* gene and a spectinomycin resistance gene (*spec*) at the opposite poles to facilitate antibiotic selection. Two microliter aliquots of the L-SCR solution of the dual-marker 1.02 Mb chromosome were mixed with a vial of HST08 competent cells (from Takara Bio, Inc.) and electroporated without arcing. One transformed colony (in reality, three neighboring colonies) was obtained using five vials of the competent cells. The agarose gel electrophoresis analysis of its L-SCR product detected the 1.02 Mb chromosome (Figure 4B). The NGS analysis of the transformed cells confirmed the SCR result (Figure 4C) and detected no spontaneous mutation. When aligning against the host genome, greater read depth was observed on the overlapping regions of the host genome and the 1.02 Mb chromosome. Because the 1.02 Mb region is derived from a genome-reduced *E. coli* strain, discontinuous regions of a total 1.02 Mb overlap over the 1.66 Mb region of the wild-type HST08 genome (Figure 4C). An important tip is that chromosomes and competent cells should be well mixed by gentle pipetting using a wide bore tip, as reported.^9^

To confirm the genetic stability in vivo as well as the physical stability in vitro, we removed the sole *Avr*II restriction site of the 1.02 Mb chromosome in the transformed strain by inserting a chloramphenicol resistance gene (*cat*) via λ-red recombination (Figure 4D). The 1.02 Mb chromosome remained episomal because it was detected from the modified strain (Figure 4D). The original and modified 1.02 Mb chromosomes were incubated in SCR mixtures with or without *Avr*II. While the original chromosome disappeared in the presence of *Avr*II, the modified Δ*Avr*II chromosome was supercoiled regardless of *Avr*II (Figure 4D).

We repeated the 1 Mb L-SCR and electroporation experiments. To increase the DNA amount, we prepared agarose plugs using 4 × 10^9^ cells. In the first trial, we got 9 hit colonies using 5 vials of competent cells. All colonies were confirmed to have the dual selection markers at the top and bottom and two PCR-checkable regions on the left and right sides of the 1.02 Mb chromosome (Figure 4C). The next trial using the same plugs yielded 4 hit colonies using 5 vials (colony number: 1, 1, 1, 1, 0). We changed the L-SCR protocol from 30 °C for 3–5 h to 24 °C for 14 h because the agarose plugs contain 4× more DNA than normal plugs. The last trial using the same plugs yielded 34 hit colonies using 6 vials (colony number: 18, 0, 7, 7, 1, 1). All hit colonies were positive by PCR. We newly prepared another plug and repeated the experiment (Figure S2). When L-SCR is successful, the L-SCR reaction solution is less viscous. The photo of 19 transformed colonies on a single selection agar plate and the result of their colony PCR check were shown (Figure S2). Thus, 1 Mb electroporation was reproducibly and routinely achieved with the optimized protocol.

L-SCR is superior to the anion-exchange column purification method^9^ in some points. Agarose plugs can be stored for months and is usable for L-SCR on demand, whereas the purified DNA solution should be prepared at the time of use (empirically, within a week). Also, purified DNA solutions are usually highly viscous. While a large volume of fresh liquid culture (750 mL) is required in the BAC purification protocol, agarose plugs can be made by scraping cells grown on agar plates. Furthermore, the BAC purification method was limited to up to 1.12 Mb chromosomes. To test even larger chromosomes, we needed a >1 Mb chromosome which can be maintained as an extra chromosome in *E. coli* cloning cells. Otherwise, it is difficult to find transformed colonies. In the next section, we sought a way to improve the genetic stability of the 1.12 Mb chromosome.

### Constructing a Portable Megabase-Sized Chromosome

**Figure 5:**
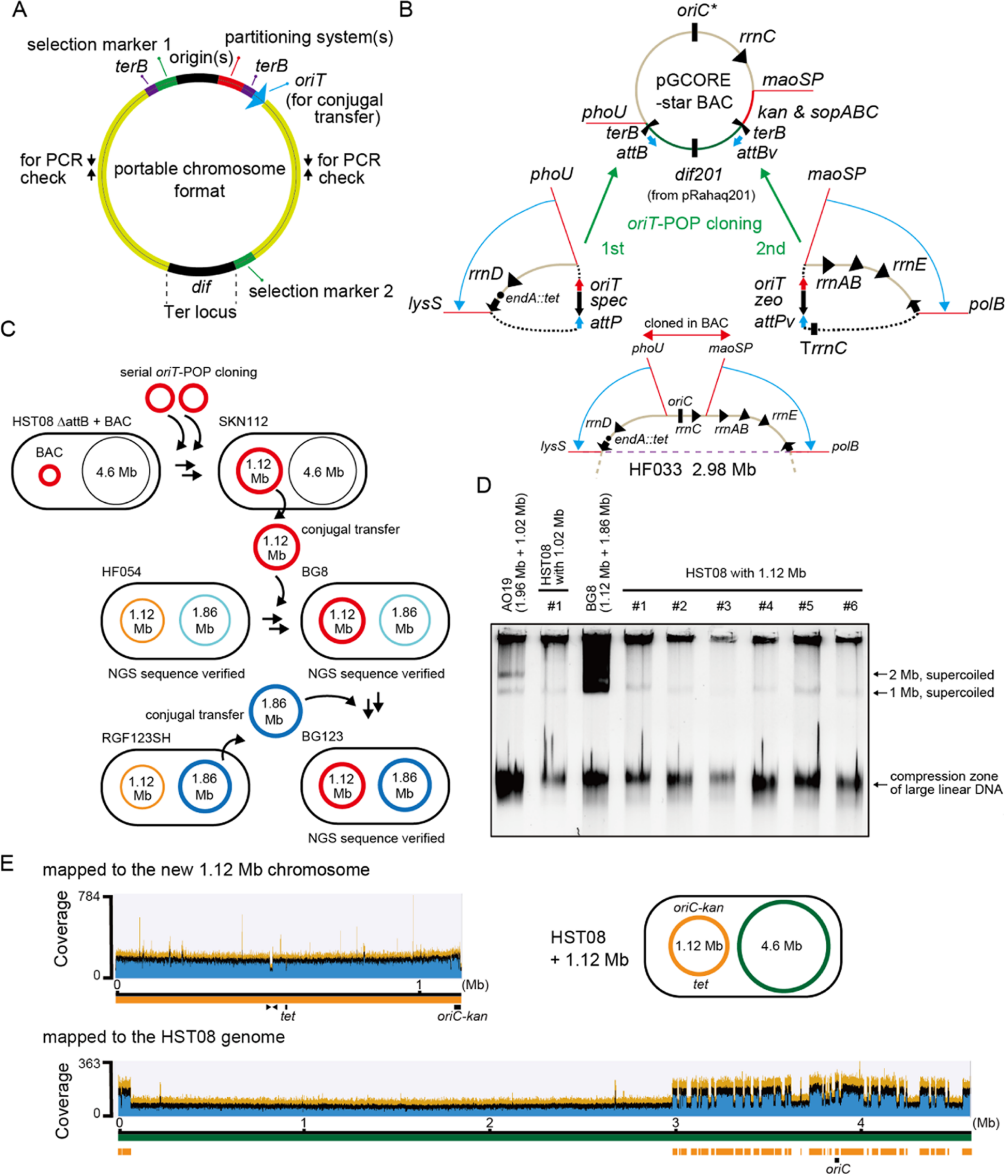
Portable chromosome for genome swap. (A) Portable chromosome format. (B) Procedure for the assembly of a portable 1.12 Mb chromosome in HST08 Δ*attB* using the pGCORE-star BAC vector via the two-step *oriT*-POP cloning from HF033-derived strains. The *oriT*-mediated transfer and circularization of the left and the right halves of the 1.12 Mb region are indicated with blue arrows and dotted lines, respectively. The HK022 integrase-mediated *attB-attP* integration is indicated with green arrows. The *attBv* and *attPv* pair is a variant of the wild-type pair *attB* and *attP*. The developed chromosome confers resistance to kanamycin, spectinomycin, zeocin, and tetracycline. (C) Procedure for the genome swap via the conjugation-based methods. Because RecA-deficient strains of *E. coli* were used, circular chromosomes were transferred as a circular chromosome upon conjugation. The whole genome sequences of HF054, BG8, and BG123 strains were verified by NGS. (D) L-SCR products of the 1.12 Mb chromosome of BG8 were introduced into *E. coli* HST08 cells via electroporation. Agarose gel electrophoresis analysis (0.3% SeaKem Gold gel, 0.5 × TBE, 40 V 90 min) of the SCR products from BG8 and the transformed HST08 clones was performed to detect the intact 1.12 Mb chromosome in the transformed HST08 clones. (E) NGS analysis of one the HST08 clones carrying the 1.12 Mb chromosome for confirming a full-length 1.12 Mb chromosome in HST08. Mapping to the HST08 reference genome showed discontinuous heaps of the duplicated regions (in total 1.12 Mb). The *oriC*, *kan*, and *tet* loci and the colony PCR site (indicated with arrows) are shown on the maps.

As mentioned in the introduction, the 1.12 Mb chromosome of the three-partite genome *E. coli* strain (1.12, 0.84, and 1.02 Mb) was not portable by electroporation nor bacterial conjugation.^9^ In what format should a portable megabase-sized chromosome be constructed? We invented a portable chromosome format (PCF) (Figure 5A). A portable chromosome should have proper replication origin(s) and chromosome partitioning system(s), at least two selection marker genes at the opposite poles, and a chromosome terminal (Ter)^46^ region located at the opposite side of the origin(s). To validate our idea, we tried to clone of the 1.12 Mb region into a BAC vector via the *oriT*-POP cloning method that was employed to assemble a conjugally transferable 1 Mb chromosome.^31^

The 1.12 Mb region carries the *oriC* region and five ribosomal RNA operons (*rrnABCDE*) as well as many other housekeeping genes (Figure 5B). A new 1.12 Mb chromosome was developed by assembling three pieces via *oriT*-POP cloning in HST08 Δ*attB*^HK022^.^31^ The 30 kb core region containing the *oriC* and the *rrnC* operon constitutes the BAC vector together with the *sopABC* genes for chromosome partitioning, a kanamycin resistance gene (*kan*) for selection, two *terB* sequences^47^ to prevent rolling circle replication, two *attB* sequences^31^ as cloning sites, and an artificial mini-Ter macrodomain^46^ containing *matS* and KOPS sequences^48^ inserted in the 8 kb region around the *dif* sequence of pRahaq201 derived from *Rahnella aquatilis*.^31,49^ The developed 42 kb minichromosome, pGCORE-star (Figure 5B), was stable in HST08 and had a spontaneous mutation in the *oriC* (oriC*). In the oriC* variant, the GATC methylation site #2^50^ is changed to TATC, which sometimes enriches during RCR amplification. The left region (from after *phoU* until after *lysS*) and then the right region (from after *maoSP* until after *polB*) of the 1.12 Mb region were cloned into pGCORE-star from the 2.98 Mb genome strain HF033 (*endA::tet*) (Figures 5B and S3A,B). To facilitate the NGS analysis of the reconstituted 1.12 Mb chromosome (Figure S3B), this chromosome was transferred to a bipartite-genome strain HF054 (1.12 Mb + 1.86 Mb) via conjugation (Figure S4A). We obtained a new bipartite-genome strain BG8 that is controlled by the reconstituted and sequence-verified 1.12 Mb chromosome and the original 1.86 Mb chromosome (Figures 5C and S4B–E). The NGS result is summarized in Figure S4, while chromosome map and sequence data are provided in the Supporting ZIP file. Since the tetracycline resistance gene (*tet*) in the left region is located at the opposite side of the *kan* gene on the 1.12 Mb chromosome, it obeys our PCF (Figure 5A). To allow antibiotic selection at high concentrations, these marker genes were expressed from strong artificial promoters.^31^

The new 1.12 Mb chromosome from BG8 cells was supercoiled by L-SCR. After electroporation using HST08 electrocompetent cells, six transformants were obtained from one vial of electrocompetent cells, while no transformant was obtained from the other four cuvettes. Interestingly, this hit cuvette was once arced at 1600 V without sparks and used for the second electroporation at 1400 V without arcing. The agarose gel electrophoresis analysis of SCR products detected the 1.12 chromosome in the six transformants (Figures 5D and S5). The NGS analysis of one of the six transformants confirmed the SCR result (Figure 5E). Thus, the new 1.12 Mb chromosome is portable. It also meant that a full set of portable split-chromosomes was obtained. We then prepared L-SCR mixtures using 2 μL aliquots of DNA solution extracted from agarose plugs made of 4 × 10^9^ BG8 cells to increase the DNA concentration (Table 1). In one experiment, 10 μL aliquots of the L-SCR solutions were mixed with 250 μL of competent cells, and 9 hit colonies were obtained using 10 vials (10 × 50 μL) of competent cells (Figure S6A). All hit colonies were positive by PCR and by dual antibiotic selection (Figure S6A). Four additional colonies that appeared after 2 days of incubation were also positive (Figure S6A). In another experiment, we obtained 49 hit colonies using 18 vials of competent cells (Figure S6B). Again, 26 additional colonies appeared after 2 days of incubation, and they all were positive (Figure S6B). We obtained only two false-positive colonies, indicating that the dual antibiotic selection was highly effective. Among the 75 clones, 48 were derived from three selective agar plates. For them, 2 μL aliquots of the L-SCR solutions were mixed with each vial of competent cells. The way of mixing DNA and cells seemed important. Under the optimal condition, four colonies per vial were obtained on average.

As for the 1.86 Mb chromosome of BG8 cells, we obtained no transformant. On the other hand, via conjugation, the 1.86 Mb chromosome of BG8 was replaced by the 1.86 Mb chromosome of RGF123SH, another bipartite-genome strain (Figure 5C). The obtained bipartite-genome strain BG123 (Figures 5C and S7A–C) has experienced a two-step complete genome swap from HF054. Future studies would increase the electroporation efficiency and stabilize a 2 Mb extra chromosome in *E. coli* cells.

## Materials and Methods

### Agarose Plugs

Agarose plugs were prepared using a new protocol slightly modified from the previous one.^9^ In principle, cells were incubated on LB agar plates and harvested. A cell pellet of 10^10^*E. coli* cells was suspended in 0.5 mL of 50 mM ethylenediaminetetraacetic acid (EDTA) (pH 8.0) and well-vortexed. As for *V. natriegens* Vmax (Codex DNA, Inc.) cells, the EDTA solution was diluted with the same volume of 2× V2 salt (final 204 mM NaCl, 4.2 mM KCl, 23.14 mM MgCl_2_) to avoid cell lysis. Low melting point agarose solutions (1.5%) prepared using PrimeGel Agarose LMT 1–20 K (Takara Bio, Inc.) and 50 mM EDTA (pH 8.0) were preheated at 45 °C. Briefly, 0.5 mL aliquots of the cell solution and the agarose solution were mixed by vortexing and immediately poured into 10 molds. Agarose plugs were cooled for >1 h in the refrigerator. One agarose plug gel is about 100 μL in volume and contains about 10^9^ cells. Each agarose plug gel was incubated with lysozyme in 1 mL of 50 mM Tris–HCl (pH 7.5) in a 2 mL tube for a few hours at 35 °C with shaking at 300 rpm using Eppendorf ThermoMixer C. The plugs were then incubated in 0.3 mL of a lysis buffer [25 U/mL Proteinase K, 1% sodium lauryl sarcosine, 0.5 M EDTA (pH 9.0)] overnight (for 16–20 h) at 50 °C with shaking at 300 rpm. The plugs were then washed with 1 mL of a wash buffer [50 mM EDTA (pH 9.5)] for 1 h on ice and with 1 mL of the wash buffer supplemented with phenylmethylsulfonyl fluoride (PMSF) (1 mM final) for another hour on ice. PMSF was removed by washing the plugs with 1 mL of Tris–EDTA (pH 8.0). The washed plugs were preserved in 1 mL of Tris–EDTA (pH 8.0) in the refrigerator for up to several months. A small block of an agarose plug (about 10^8^ cells) was digested using 0.5 μL of Thermostable β-agarase (Nippon Gene) at 65 °C for 10 min to elute the DNA molecules and was then cooled on ice.

### Replication-Cycle Reaction (RCR)

The RCR reagents were modified from the original receipt^39^ for replicating megabase-sized chromosomes. In addition to the supplementation of RecG, RecJ, *Exo*III, and λ phage DNA to the reaction,^31^ dimethyl sulfoxide (DMSO) and dextran were added at the final concentrations of 0.1 and 2.5%, respectively. The new lot of RCR enzyme mix (0004Y) contains 2.8× LigA compared to the original one.^39^ Reaction mixtures were incubated for 10–40 cycles at 37 °C for 30 s and 22 °C for 60 min. After the RCR incubation, the reaction mixtures with 1× enzymes were diluted by five times with 1.25× RCR buffer with or without *Dpn*I (final 0.4 U/μL) and incubated for an additional hour at 30 °C. The reaction mixtures with 0.5× enzymes were diluted by two times with 1.4× RCR buffer with/without *Dpn*I and incubated at 22 °C. This second incubation step finalizes the last chromosome replication cycle. *Dpn*I cleaves methylated and hemi-methylated GATC sites during the second incubation step.

### Supercoiling and Repair Reaction (SCR)

The 10× SCR mix includes 1 mg/mL bovine serum albumin (BSA) (Roche), 140 nM LigA (Takara Bio or NIPPON GENE), 170 nM of *Pol*I (Takara Bio), 200 U/μL *Exo*III (Takara Bio), and 250 nM of GyrAB (Takara Bio) and was diluted using a dilution buffer [final concentrations: 10% glycerol, 20 mM Tris–HCl (pH 7.5), 0.1 mg/mL BSA, 8 mM DTT, 10 mM Mg(OAc)_2_, and 125 mM KOAc]. The RCR buffer^39^ is the SCR buffer. The 10× L-SCR mix was prepared by omitting KOAc and DTT from the dilution buffer. The L-SCR buffer is also devoid of KOAc and DTT. Usually, 1 μL aliquot of eluted DNA solutions was incubated in a 10 μL reaction volume in a PCR tube using ProFlex PCR System (Applied Biosystems). The *Dpn*I reaction of SCR products was performed in the same manner as for RCR products. Electroporation was performed as described^9^ using ELEPO21 (Nepa Gene).

### Agarose Gel Electrophoresis

The conventional agarose gel electrophoresis analyses were performed essentially as reported.^31^ For megabase-sized chromosomes, 0.3% gels were made using SeaKem Gold agarose (Lonza)^42^ and run for 1–2 h at a 40 V constant voltage in 0.5× TBE buffer. The PFGE analysis was performed as reported.^9^ DNA ladder markers were Nippon Gene Marker 3 and Gene Ladder 100, and ExcelBand 1 KB (0.25–10 kb) DNA Ladder DM3100 (SMO). The gels were stained with dsGreen (Funakoshi) and scanned by a Typhoon FLA 9500 (GE Healthcare). Images were developed and edited using NIH ImageJ.

### Plasmids

Plasmids were constructed essentially in the same manner as described.^9,31^ For constructing pGCORE-star, we used Q5 High-fidelity DNA polymerase (NEB) and RA-RCR^39^ (equivalent to the OriCiro Cell-Free Cloning System of OriCiro Genomics, Inc.). pBAD-traRP4min-pac was developed by inserting a puromycin *N*-acetyltransferase gene (*pac*) under control of the EM7 promoter into the *Pst*I site inside the ampicillin resistance gene (*bla*).

### Chromosome and Cell Engineering

Chromosomes were modified via λ-red recombination using PCR products and several helper plasmids (Table 2), as reported previously.^9^ Since the chromosomal integration of an *oriT* cassette and large DNA cassettes was very inefficient in DGF-298W Δ*recAX* strains, we expressed the native *recAX* operon from a helper plasmid. New chromosomes were assembled via dual Flp-POP cloning method or via *oriT*-POP cloning method^31^ using a new helper plasmid pKD46-int. The dual Flp-POP cloning method was updated from Flp-POP cloning method^31^ for the nearly simultaneous pop-out and cloning of two genomic regions. To achieve this, expanded combinations of antibiotic resistance genes were employed. After induction of the flippase and HK022 integrase from pMW118gent-flp-int,^9^ the two genomic regions were each circularized and cloned into the two HK022 *attB* sites (*attB* and *attBv*) in the BAC vector pVtu9xV2, while the main chromosome gained the full-length *cat* and *spec* genes. Spectinomycin resistance colonies were subjected to chloramphenicol selection, and vice versa, to finally obtain bipartite-genome strains. The portable 1.12 Mb chromosome was constructed in HST08 Δ*attB* carrying pGCORE-star via the two-step *oriT*-POP cloning^31^ of the left and right halves of the 1.12 Mb region. Because the insertion of the original *attPv-zeo-oriT* cassette^31^ between the *polB* and *leuD* genes were not successful, we added an *rrnC* terminator in front of the *attPv* sequence. pGCORE-star has two cloning sites (*attB* and *attBv*).^31^ We realized that HF033 as well as DGF-298W had experienced an occasional genomic inversion between the *rrnD* and *rrnE* operons^51^ during the establishment of DGF-298W from W3110. Unlike W3110, DGF-298W and its descendants have a typical configuration of the ribosomal RNA operons in the genome.^51^ The left half was first cloned into *attB* site to develop the half-sized chromosome. Then the right half was cloned into the *attBv* site of the half-sized chromosome to develop the full-length chromosome. In addition to the combination of pGCORE-star and HST08 Δ*attB*, we also examined pGCORE-wt carrying the wild-type *oriC* sequence and HST08 Δ*attB* Δ*tus* deficient in the replication fork trap system, as demonstrated in Supporting Figure S3. We chose the original combination because two of the authors individually succeeded in the construction. Bacterial conjugation was performed as described^9,31^ but with a choice of two helper plasmids carrying either of the ampicillin and puromycin selection markers. By an unknown reason, DGF-298W derivatives exhibited a native resistance to puromycin. A strain list is provided (Table 3). The sequence information of DNA cassettes and homology arms are provided (Supporting Tables S1 and S2).

**Table 2:** List of Helper Plasmids and BAC Vectors^a^

name	description
pKD46^52^	*t*_s_, λ-red recombination
pMW118-gba^31^	λ-red recombination
pMW118-g’ba^31^	λ-red recombination, Δ*gam*, “curable”
pMW118-recAX-g’ba	λ-red recombination, Δ*gam*, *E. colirecAX* expression, hardly “curable”
pMW118gent	*gent*, “curable”
pKD46-int	*t*_s_, HK022 integrase expression
pMW118gent-flp-int^9^	flippase and integrase expression, “curable”
pBAD-traRP4min^31^	RP4 *tra* expression, Δ*oriT*
pBAD-traRP4min-pac	RP4 *tra* expression, Δ*oriT*, *bla::pac*
pOri2spec-recAX^31^	*E. colirecAX* expression, “curable”
pVtu9xT^31^	*V. tubiashiiori2*-based BAC vector with *oriT*
pVtu9xF^9^	*V. tubiashiiori2*-based BAC vector without *oriT*
pVtu9xV2	*V. tubiashiiori2*-based BAC vector, pVtudif2^31^ derivative lacking *crtS* and 1× *parS2*
pGCORE-star	BAC vector for developing a portable 1.12 Mb chromosome
pGCORE-wt	pGCORE-star variant with the wild-type *oriC*

a *t*_s_ indicates that the plasmid is temperature sensitive. “Curable” indicates that the indicated helper plasmid is easily curable in DGF-298W-derived strains.

**Table 3:** List of *E. coli* and *V. natriegens* Strains^a^

name	notes
DGF-298W^24^	*E. coli* K-12 W3110 derivative DGF-298WΔ100:revΔ234:SC
RGF008C^9^	DGF-298W Δ*sacB-cat* Δ*terFIJ* Δ*recAX*
RGF123SH	RGF123-derived,^9^ [Chr^Ori^ and Chr^LR+Ter^ (pVtu9xT) with RCR*ori-kan::spec-hyg*] with pBAD-traRP4min
HF014	DGF-298W Δ*sacB-cat* Δ*terFIJ* Δ*tus kan*-*oriC*@*ybgL-gltA hyg*-*oriC*@*glpC-menF endA::tet*
HF025	HST08 *tus::zeo* with a 530 kb plasmid^31^
HF033^9^	RGF008C derivative with *endA::tet*
HF053^9^	RGF008C-derived, *endA::tet*, [Chr^Ori+LR^ and Chr^Ter^ (pVtu9xF)]
HF054^9^	RGF008C-derived, *endA::tet*, [Chr^Ori^ and Chr^LR+Ter^ (pVtu9xF)]
HF055	HF033 derivative with *kan*@*ybgL-gltA*
AO13	HST08 with pMSR227 (205 kb)^39^
AO19	HF053-derived, [Chr^Ori+LR^ and Chr^Ter^ with *spec*@*dif*]
HST08	F–, *endA1, supE44, thi-1, recA1, relA1, gyrA96, phoA*, Φ80d*lacZ*ΔM15, Δ(*lacZYA-argF*)U169, Δ(*mrr-hsdRMS-mcrBC*), Δ*mcrA*, λ–
HST08 Δ*attB*^31^	no *attB*^HK022^ site
HST08 Δ*attB* Δ*tus*^31^	used for cloning genome regions with *terB* sequences
YST01^9^	HST08 carrying Chr^LR^ originated from RGF152^9^
YST03^9^	HST08 carrying Chr^Ter^ originated from HF053
SKN112	HST08 Δ*attB* carrying portable Chr^Ori^, pKD46-int, pBAD-traRP4min-pac, and pGCORE-star
BG5 (no. 1)	HF054-derived, [portable ChrOri and pGCORE-star from SKN112 and Chr^LR+Ter^]
BG7	BG5-derived, [portable ChrOri and pGCORE-star and Chr^LR+Ter^ with *cat*@*murJ-rne*]
BG8	BG7-derived, [portable Chr^Ori^ and Chr^LR+Ter^ with *cat*@*murJ-rne*]
BG123	BG8-derived, [portable Chr^Ori^ and Chr^LR+Ter^ from RGF123SH]
YGF012	RGF008C-derived, [Chr (1.67 Mb) and Chr (1.31 Mb using pVtu9xV2)]
YGF017	RGF008C-derived, [Chr (2.14 Mb) and Chr (0.84 Mb using pVtu9xV2)]
V. natriegens^53^ Vmax Express	a clean genome strain of V. natriegens (Codex DNA, Inc.)

a Abbreviations of split-chromosomes according to the previous study:^9^ Chr^Ori^, Chr^LR^, Chr^Ter^, Chr^Ori+LR^, and Chr^LR+Ter^ denote 1.12, 0.84, 1.02, 1.96, and 1.86 Mb chromosomes, respectively.

### NGS

The NGS analyses of some genomes, chromosomes, and plasmids were performed using iSeq 100 (Illumina) and NEBNext Ultra II FS DNA Library Prep with Sample Purification Beads and Multiplex Oligos for Illumina (NEB). The FASTQ data were analyzed using Geneious Prime (Biomatters Ltd.). DNA sequence and map data were edited using SnapGene (GSL Biotech LLC) and provided as a Supporting ZIP file. The draft genome sequences of HST08 and DGF-298W were assembled previously.^31^

## Supporting Information Available

Developing bipartite-genome strains via dual Flp-POP cloning (Figure S1); electroporation of HST08 with a 1.02 Mb chromosome prepared by L-SCR (Figure S2); assembling a portable 1.12 Mb chromosome (Figure S3); portable chromosome for partial genome swap (Figure S4); detecting the portable 1.12 Mb chromosome (Figure S5); electroporation of HST08 with a 1.12 Mb chromosome prepared by L-SCR (Figure S6); two-step genome swap (Figure S7); the sequences of the genetic cassettes used in this study (Table S1); the sequences of the homology arms used in the λ red recombination experiments (Table S2) (PDF)
Plasmid and chromosome maps and sequences (ZIP)

## Abbreviations

RCR: replication-cycle reaction
SCR: supercoiling and repair reaction
PCR: polymerase chain reaction
NGS: next-generation sequencing
TBE: Tris borate-EDTA
PFGE: pulsed-field gel electrophoresis
BAC: bacterial artificial chromosome
