# Supplementary material for: Enzymatic Supercoiling of Bacterial Chromosomes Facilitates Genome Manipulation
Source: ACS Synth Biol. 2022 Aug 23;11(9):3088–99. doi: 10.1021/acssynbio.2c00353 (PMC9486964; doi:10.1021/acssynbio.2c00353)
Supplement: Supplementary file 1 — sb2c00353_si_001.pdf [file sb2c00353_si_001.pdf]

Enzymatic supercoiling of bacterial chromosomes facilitates genome manipulation

Hironobu Fujita,† Ayane Osaku,† Yuto Sakane,[1] Koki Yoshida, Kayoko Yamada,[2] Seia Nara,[2] Takahito Mukai* and Masayuki Su'etsugu*

Department of Life Science, College of Science, Rikkyo University, Tokyo 171-8501, Japan.

[1]Present address: Department of Biological Sciences, Graduate School of Science, The University of Tokyo, Tokyo, 113-0032, Japan. [2]Present address: OriCiro Genomics, Inc., Tokyo 113-8485, Japan.

Supplementary

Table S1. The sequences of the genetic cassettes used in this study.

```
>50-TrrnC-zeo-PEM7-oriT>-BGHfwd-Cpf1s1 for oriT-POP cloning
GACGCAGTTCAACTCAGCTGTACACATCAAAGCTTTCGACGAAATGCATGcaaaaaaaatccttagctttcgctaag
gatctgcaTCAGTCCTGCTCCTCGGCCACGAAGTGCACGCAGTTGCCGGCCGGGTCGCGCAGGGCGAACTCCCGCCC
CCACGGCTGCTCGCCGATCTCGGTCATGGCCGGCCCGGAGGCGTCCCGGAAGTTCGTGGACACGACCTCCGACCACT
CGGCGTACAGCTCGTCCAGGCCGCGCACCCACACCCAGGCCAGGGTGTTGTCCGGCACCACCTGGTCCTGGACCGCG
CTGATGAACAGGGTCACGTCGTCCCGGACCACACCGGCGAAGTCGTCCTCCACGAAGTCCCGGGAGAACCCGAGCCG
GTCGGTCCAGAACTCGACCGCTCCGGCGACGTCGCGCGCGGTGAGCACCGGAACGGCACTGGTCAACTTGGCCATGG
TTTAGTTCCTCACCTTGTCGTATTATACTATGCCGATATACTATGCCGATGATTAATTGTCAACACGTGCTGATCAG
ATCCGCAGCGCTTTTCCGCTGCATAACCCTGCTTCGGGGTCATTATAGCGATTTTTTCGGTATATCCATCCTTTTTC
GCACGATATACAGGATTTTGCCAAAGGGTTCGTGTAGACTTTCCTTGgtgtatccaacggcgtcagccgggcaggat
aggtgaagtaggcccacccgcgagcgggtgttccttcttcactgtcccttattcGCACCTGGCGGTGCTCAACGGGA
ATCCTGCTCTGCGAGGCTGGCCGGCTGGCAACTAGAAGGCACAGCCTTTAGAGAAGTCATTTAATAAGGCCACT
```

```
>Cpf1s3-attP-Tlpp-spec-PEM7-oriT>-BGHfwd for oriT-POP cloning
CTACTAATACAAAAGGCTCTAGACCAAAGGTCACTAATACTATCTAAGTAGTTGATTCATAGTGACTGGATATGTTG
CGTTTTGTCGCATTATGTAGTCTATCATTTAACCACAGATTAGTGTAATGCGATGATTTTTAAGTGATTAATGTTAT
TTTGTCATCCTTTAGGTGAATAAGTTGTATATTTAAAATCTCTTTAATTATCAGTAAATTAATGTAAGTAGGTCATT
ATTAGTCAAAATAAAATCATTTGcaaaaaaaatggcgcacaatgtgcgccaTTATTTGCCGACTACCTTGGTGATCT
CGCCTTTCACGTAGTGGACAAATTCTTCCAACTGATCTGCGCGCGAGGCCAAGCGATCTTCTTCTTGTCCAAGATAA
GCCTGTCTAGCTTCAAGTATGACGGGCTGATACTGGGCCGGCAGGCGCTCCATTGCCCAGTCGGCAGCGACATCCTT
CGGCGCGATTTTGCCGGTTACTGCGCTGTACCAAATGCGGGACAACGTAAGCACTACATTTCGCTCATCGCCAGCCC
AGTCGGGCGGCGAGTTCCATAGCGTTAAGGTTTCATTTAGCGCCTCAAATAGATCCTGTTCAGGAACCGGATCAAAG
AGTTCCTCCGCCGCTGGACCTACCAAGGCAACGCTATGTTCTCTTGCTTTTGTCAGCAAGATAGCCAGATCAATGTC
GATCGTGGCTGGCTCGAAGATACCTGCAAGAATGTCATTGCGCTGCCATTCTCCAAATTGCAGTTCGCGCTTAGCTG
GATAACGCCACGGAATGATGTCGTCGTGCACAACAATGGTGACTTCTACAGCGCGGAGAATCTCGCTCTCTCCAGGG
GAAGCCGAAGTTTCCAAAAGGTCGTTGATCAAAGCTCGCCGCGTTGTTTCATCAAGCCTTACGGTCACCGTAACCAG
CAAATCAATATCACTGTGTGGCTTCAGGCCGCCATCCACTGCGGAGCCGTACAAATGTACGGCCAGCAACGTCGGTT
CGAGATGGCGCTCGATGACGCCAACTACCTCTGATAGTTGAGTCGATACTTCGGCGATCACCGCTTCCCTCATGGTT
TAGTTCCTCACCTTGTCGTATTATACTATGCCGATATACTATGCCGATGATTAATTGTCAACACGTGCTGATCAGAT
CCGCAGCGCTTTTCCGCTGCATAACCCTGCTTCGGGGTCATTATAGCGATTTTTTCGGTATATCCATCCTTTTTCGC
ACGATATACAGGATTTTGCCAAAGGGTTCGTGTAGACTTTCCTTGgtgtatccaacggcgtcagccgggcaggatag
gtgaagtaggcccacccgcgagcgggtgttccttcttcactgtcccttattcGCACCTGGCGGTGCTCAACGGGAAT
CCTGCTCTGCGAGGCTGGCCGGCTGGCAACTAGAAGGCACAG
```

>50-TrrnC-spec-PEM7-oriT>-BGHfwd-Cpf1s1 for oriT-POP cloning

GACGCAGTTCAACTCAGCTGTACACATCAAAGCTTTCGACGAAATGCATGcaaaaaaaatccttagctttcgctaag
gatctgcaTTATTTGCCGACTACCTTGGTGATCTCGCCTTTCACGTAGTGGACAAATTCTTCCAACTGATCTGCGCG
CGAGGCCAAGCGATCTTCTTCTTGTCCAAGATAAGCCTGTCTAGCTTCAAGTATGACGGGCTGATACTGGGCCGGCA
GGCGCTCCATTGCCCAGTCGGCAGCGACATCCTTCGGCGCGATTTTGCCGGTTACTGCGCTGTACCAAATGCGGGAC
AACGTAAGCACTACATTTCGCTCATCGCCAGCCCAGTCGGGCGGCGAGTTCCATAGCGTTAAGGTTTCATTTAGCGC
CTCAAATAGATCCTGTTCAGGAACCGGATCAAAGAGTTCCTCCGCCGCTGGACCTACCAAGGCAACGCTATGTTCTC
TTGCTTTTGTCAGCAAGATAGCCAGATCAATGTCGATCGTGGCTGGCTCGAAGATACCTGCAAGAATGTCATTGCGC
TGCCATTCTCCAAATTGCAGTTCGCGCTTAGCTGGATAACGCCACGGAATGATGTCGTCGTGCACAACAATGGTGAC
TTCTACAGCGCGGAGAATCTCGCTCTCTCCAGGGGAAGCCGAAGTTTCCAAAAGGTCGTTGATCAAAGCTCGCCGCG
TTGTTTCATCAAGCCTTACGGTCACCGTAACCAGCAAATCAATATCACTGTGTGGCTTCAGGCCGCCATCCACTGCG
GAGCCGTACAAATGTACGGCCAGCAACGTCGGTTCGAGATGGCGCTCGATGACGCCAACTACCTCTGATAGTTGAGT
CGATACTTCGGCGATCACCGCTTCCCTCATGGTTTAGTTCCTCACCTTGTCGTATTATACTATGCCGATATACTATG
CCGATGATTAATTGTCAACACGTGCTGATCAGATCCGCAGCGCTTTTCCGCTGCATAACCCTGCTTCGGGGTCATTA
TAGCGATTTTTTCGGTATATCCATCCTTTTTCGCACGATATACAGGATTTTGCCAAAGGGTTCGTGTAGACTTTCCT
TGgtgtatccaacggcgtcagccgggcaggataggtgaagtaggcccacccgcgagcgggtgttccttcttcactgt
cccttattcGCACCTGGCGGTGCTCAACGGGAATCCTGCTCTGCGAGGCTGGCCGGCTGGCAACTAGAAGGCACAGC
CTTTAGAGAAGTCATTTAATAAGGCCACT

>TrrnC-Cpf1s3-attPv-Tlpp-zeo-PEM7-oriT>-BGHfwd for oriT-POP cloning

ATTTCCGTCGAGGCAAAAAAAATCCTTAGCTTTCGCTAAGGATCTACTAATACAAAAGGCTCTAGACCAAAGGTCAC
TAATACTATCTAAGTAGTTGATTCATAGTGACTGGATATGTTGCGTTTTGTCGCATTATGTAGTCTATCATTTAACC
ACAGATTAGTGTAATGCGATGATTTTTAAGTGATTAATGTTATTTTGTCATCCTTTTACAATGTAAGTTGTATATTT
AAAATCTCTTTAATTATCAGTAAATTAATGTAAGTAGGTCATTATTAGTCAAAATAAAATCATTTGcaaaaaaatg
gcgcacaatgtgcgccaTTATCAGTCCTGCTCCTCGGCCACGAAGTGCACGCAGTTGCCGGCCGGGTCGCGCAGGGC
GAACTCCCGCCCCCACGGCTGCTCGCCGATCTCGGTCATGGCCGGCCCGGAGGCGTCCCGGAAGTTCGTGGACACGA
CCTCCGACCACTCGGCGTACAGCTCGTCCAGGCCGCGCACCCACACCCAGGCCAGGGTGTTGTCCGGCACCACCTGG
TCCTGGACCGCGCTGATGAACAGGGTCACGTCGTCCCGGACCACACCGGCGAAGTCGTCCTCCACGAAGTCCCGGGA
GAACCCGAGCCGGTCGGTCCAGAACTCGACCGCTCCGGCGACGTCGCGCGCGGTGAGCACCGGAACGGCACTGGTCA
ACTTGGCCATGGTTTAGTTCCTCACCTTGTCGTATTATACTATGCCGATATACTATGCCGATGATTAATTGTCAACA
CGTGCTGATCAGATCCGCAGCGCTTTTCCGCTGCATAACCCTGCTTCGGGGTCATTATAGCGATTTTTTCGGTATAT
CCATCCTTTTTCGCACGATATACAGGATTTTGCCAAAGGGTTCGTGTAGACTTTCCTTGgtgtatccaacggcgtca
gccgggcaggataggtgaagtaggcccacccgcgagcgggtgttccttcttcactgtcccttattcGCACCTGGCGG
TGCTCAACGGGAATCCTGCTCTGCGAGGCTGGCCGGCTGGCAACTAGAAGGCACAG

>dif-spec for modifying dif

attcttccttggtttatattggtgcgcataatgtatattatgttaaatCGGATCTGATCAGCACGTGTTGACAATTA
ATCATCGGCATAGTATATCGGCATAGTATAATACGACAAGGTGAGGAACTAAACCATGAGGGAAGCGGTGATCGCCG
AAGTATCGACTCAACTATCAGAGGTAGTTGGCGTCATCGAGCGCCATCTCGAACCGACGTTGCTGGCCGTACATTTG
TACGGCTCCGCAGTGGATGGCGGCCTGAAGCCACACAGTGATATTGATTTGCTGGTTACGGTGACCGTAAGGCTTGA
TGAAACAACGCGGCGAGCTTTGATCAACGACCTTTTGGAAACTTCGGCTTCCCCTGGAGAGAGCGAGATTCTCCGCG
CTGTAGAAGTCACCATTGTTGTGCACGACGACATCATTCCGTGGCGTTATCCAGCTAAGCGCGAACTGCAATTTGGA
GAATGGCAGCGCAATGACATTCTTGCAGGTATCTTCGAGCCAGCCACGATCGACATTGATCTGGCTATCTTGCTGAC
AAAAGCAAGAGAACATAGCGTTGCCTTGGTAGGTCCAGCGGCGGAGGAACTCTTTGATCCGGTTCCTGAACAGGATC
TATTTGAGGCGCTAAATGAAACCTTAACGCTATGGAACTCGCCGCCCGACTGGGCTGGCGATGAGCGAAATGTAGTG
CTTACGTTGTCCCGCATTTGGTACAGCGCAGTAACCGGCAAAATCGCGCCGAAGGATGTCGCTGCCGACTGGGCAAT
GGAGCGCCTGCCGGCCCAGTATCAGCCCGTCATACTTGAAGCTAGACAGGCTTATCTTGGACAAGAAGAAGATCGCT
TGGCCTCGCGCGCAGATCAGTTGGAAGAATTTGTCCACTACGTGAAAGGCGAGATCACCAAGGTAGTCGGCAAATAA
tgccatgtctgagtgatgcgaagtgcttttctggtagtcgttattcgttc

>hyg-spec-TfdhF for replacing kan-Ptac’-oriC-TfdhF

aaagccacgttgtgtctcaaaatctctgatgttgcattgcacaagataaaaatatatcatcatgaacaataaaactg
tctgcttacataaacggaaaatttttttcaaaagtacttgacaacataacggataacaattgtaatagattcaatt

gtaacgtaatacaaggaacgtgttATGAAAAAACCGGAACTGACCGCAACCAGCGTTGAAAAATTTCTGATCGAAAA
ATTCGATAGCGTGAGCGATCTGATGCAGCTGAGCGAAGGTGAAGAAAGCCGTGCATTTAGCTTTGATGTTGGTGGTC
GTGGTTATGTTCTGCGTGTTAATAGCTGTGCAGATGGCTTCTATAAAGATCGTTATGTGTATCGTCATTTTGCAAGC
GCAGCACTGCCGATTCCGGAAGTTCTGGATATTGGTGAATTTAGCGAAAGCCTGACCTATTGTATTAGCCGTCGTGC
ACAGGGTGTTACCCTGCAGGATCTGCCGGAAACCGAACTGCCTGCAGTTCTGCAGCCGGTTGCCGAAGCAATGGATG
CAATTGCCGCAGCAGATCTGAGCCAGACCAGCGGTTTTGGTCCGTTTGGTCCTCAAGGTATTGGTCAGTATACCACC
TGGCGTGATTTTATCTGTGCCATTGCAGATCCGCATGTTTATCATTGGCAGACCGTTATGGATGATACCGTTAGCGC
AAGCGTTGCACAGGCACTGGATGAACTGATGCTGTGGGCAGAAGATTGTCCTGAAGTTCGTCATCTGGTTCATGCAG
ATTTTGGTAGCAATAATGTGCTGACCGATAATGGTCGTATTACCGCAGTTATTGATTGGAGCGAAGCCATGTTTGGT
GATAGCCAGTATGAAGTTGCCAACATCTTTTTTTGGCGTCCGTGGCTGGCATGTATGGAACAGCAGACCCGTTATTT
TGAACGTCGTCATCCGGAACTGGCAGGTAGTCCGCGTCTGCGTGCCTATATGCTGCGTATTGGTCTGGATCAGCTGT
ATCAGTCACTGGTTGATGGTAATTTTGATGATGCAGCATGGGCACAGGGTCGTTGTGATGCCATTGTTCGTAGCGGT
GCAGGCACCGTTGGTCGTACCCAGATTGCACGTCGTAGCGCAGCAGTTTGGACCGATGGTTGTGTTGAAGTGCTGGC
AGATAGCGGTAATCGTCGTCCGAGCACACGTCCGGATCGTGAAATGGGTGAAGCAAATTAAaCGGATCTGATCAGCA
CGTGTTGACAATTAATCATCGGCATAGTATATCGGCATAGTATAATACGACAAGGTGAGGAACTAAACCATGAGGGA
AGCGGTGATCGCCGAAGTATCGACTCAACTATCAGAGGTAGTTGGCGTCATCGAGCGCCATCTCGAACCGACGTTGC
TGGCCGTACATTTGTACGGCTCCGCAGTGGATGGCGGCCTGAAGCCACACAGTGATATTGATTTGCTGGTTACGGTG
ACCGTAAGGCTTGATGAAACAACGCGGCGAGCTTTGATCAACGACCTTTTGGAAACTTCGGCTTCCCCTGGAGAGAG
CGAGATTCTCCGCGCTGTAGAAGTCACCATTGTTGTGCACGACGACATCATTCCGTGGCGTTATCCAGCTAAGCGCG
AACTGCAATTTGGAGAATGGCAGCGCAATGACATTCTTGCAGGTATCTTCGAGCCAGCCACGATCGACATTGATCTG
GCTATCTTGCTGACAAAAGCAAGAGAACATAGCGTTGCCTTGGTAGGTCCAGCGGCGGAGGAACTCTTTGATCCGGT
TCCTGAACAGGATCTATTTGAGGCGCTAAATGAAACCTTAACGCTATGGAACTCGCCGCCCGACTGGGCTGGCGATG
AGCGAAATGTAGTGCTTACGTTGTCCCGCATTTGGTACAGCGCAGTAACCGGCAAAATCGCGCCGAAGGATGTCGCT
GCCGACTGGGCAATGGAGCGCCTGCCGGCCCAGTATCAGCCCGTCATACTTGAAGCTAGACAGGCTTATCTTGGACA
AGAAGAAGATCGCTTGGCCTCGCGCGCAGATCAGTTGGAAGAATTTGTCCACTACGTGAAAGGCGAGATCACCAAGG
TAGTCGGCAAATAAtaaCAGCTCATTTCAGAATATTTGCCTACAGCCTCCTTTCGGAGGCTGTTTTTTTAccattct
tctgccggatcttc

>Pkan-hyg-Pcat’-cat5’-FRT-Cpf1s1 for dual Flp-POP cloning

aaagccacgttgtgtctcaaaatctctgatgttgcattgcacaagataaaaatatatcatcatgaacaataaaactg
tctgcttacataaacggaaaattttttttcaaaagtacttgacaacataacggataacaattgtaatagattcaatt
gtaacgtaatacaaggaacgtgttATGAAAAAACCGGAACTGACCGCAACCAGCGTTGAAAAATTTCTGATCGAAAA
ATTCGATAGCGTGAGCGATCTGATGCAGCTGAGCGAAGGTGAAGAAAGCCGTGCATTTAGCTTTGATGTTGGTGGTC
GTGGTTATGTTCTGCGTGTTAATAGCTGTGCAGATGGCTTCTATAAAGATCGTTATGTGTATCGTCATTTTGCAAGC
GCAGCACTGCCGATTCCGGAAGTTCTGGATATTGGTGAATTTAGCGAAAGCCTGACCTATTGTATTAGCCGTCGTGC
ACAGGGTGTTACCCTGCAGGATCTGCCGGAAACCGAACTGCCTGCAGTTCTGCAGCCGGTTGCCGAAGCAATGGATG
CAATTGCCGCAGCAGATCTGAGCCAGACCAGCGGTTTTGGTCCGTTTGGTCCTCAAGGTATTGGTCAGTATACCACC
TGGCGTGATTTTATCTGTGCCATTGCAGATCCGCATGTTTATCATTGGCAGACCGTTATGGATGATACCGTTAGCGC
AAGCGTTGCACAGGCACTGGATGAACTGATGCTGTGGGCAGAAGATTGTCCTGAAGTTCGTCATCTGGTTCATGCAG
ATTTTGGTAGCAATAATGTGCTGACCGATAATGGTCGTATTACCGCAGTTATTGATTGGAGCGAAGCCATGTTTGGT
GATAGCCAGTATGAAGTTGCCAACATCTTTTTTTGGCGTCCGTGGCTGGCATGTATGGAACAGCAGACCCGTTATTT
TGAACGTCGTCATCCGGAACTGGCAGGTAGTCCGCGTCTGCGTGCCTATATGCTGCGTATTGGTCTGGATCAGCTGT
ATCAGTCACTGGTTGATGGTAATTTTGATGATGCAGCATGGGCACAGGGTCGTTGTGATGCCATTGTTCGTAGCGGT
GCAGGCACCGTTGGTCGTACCCAGATTGCACGTCGTAGCGCAGCAGTTTGGACCGATGGTTGTGTTGAAGTGCTGGC
AGATAGCGGTAATCGTCGTCCGAGCACACGTCCGGATCGTGAAATGGGTGAAGCAAATTAAtgcGCTGGCAACTAGA
AGGCACAGcgtaagaggttccaactttcaccatattgacataagatcactaccgggcgtataatttgagttatcgag
attttcaggagctaaggaagctaaaATGGAGAAAAAAATCACTGGATATACCACCGTTGATATATCCCAATGGCATC
GTAAAGAACATTTTGAGGCATTTCAGTCAGTTGCTCAATGTACCTATAACCAGACCGTTCAGCTGGATATTACGGCC
TTTTTAAAGACCGTAAAGAAAAATAAGCACAAGTTTTATCCGGCCTTTATTCACATTCTTGCCCGCCTGATGAATGC
TCATCCGGAATTCCGTATGGCAATGAAAGACGGTGAGCTGGTGATATGGGATAGTGTTCACCCTTGTTACACCGTTT
TCCATGAGCAAACTGAAACGTTTTCATCGCTCTGGAGTGAATACCACGACGATTTCCGGCAGTTTCTACACATATAT
TCGCAAGATGTGGCGTGTTACggaagttcctattctctagaaagtataggaacttcCAGGTCTTTCTCAAGCCGAcc
tttagagaagtcatttaataaggccact

>Cpf1s3-attP-FRT-cat3’-Ttrp-PEM7-tet-Tlpp-TrrnC for dual Flp-POP cloning

CTACTAATACAAAAGGCTCTAGACCAAAGGTCACTAATACTATCTAAGTAGTTGATTCATAGTGACTGGATATGTTG
CGTTTTGTCGCATTATGTAGTCTATCATTTAACCACAGATTAGTGTAATGCGATGATTTTTAAGTGATTAATGTTAT
TTTGTCATCCTTTAGGTGAATAAGTTGTATATTTAAAATCTCTTTAATTATCAGTAAATTAATGTAAGTAGGTCATT
ATTAGTCAAAATAAAATCATTTGcgctgcttttgcacgtgagGAAGTTCCTATTCTCTAGAAAGTATAGGAACTTCt
GAAAACCTGGCCTATTTCCCTAAAGGGTTTATTGAGAATATGTTTTTCGTCTCAGCCAATCCCTGGGTGAGTTTCAC
CAGTTTTGATTTAAACGTGGCCAATATGGACAACTTCTTCGCCCCCGTTTTCACCATGGGCAAATATTATACGCAAG
GCGACAAGGTGCTGATGCCGCTGGCGATTCAGGTTCATCATGCCGTtTGTGATGGCTTCCATGTCGGCAGAATGCTT
AATGAATTACAACAGTACTGCGATGAGTGGCAGGGCGGGGCGTAAtcccacagccgccagttccgctggcggcattt
tgACTGCCATGGAAAATCGATGTTCTTCGGATCTGATCAGCACGTGTTGACAATTAATCATCGGCATAGTATATCGG
CATAGTATAATACGACAAGGTGAGGAACTAAACCATGAAATCTAACAATGCGCTCATCGTCATCCTCGGCACCGTCA
CCCTGGATGCTGTAGGCATAGGCTTGGTTATGCCGGTACTGCCGGGCCTCTTGCGGGATATCGTCCATTCCGACAGC
ATCGCCAGTCACTATGGCGTGCTGCTAGCGCTATATGCGTTGATGCAATTTCTATGCGCACCCGTTCTCGGAGCACT
GTCCGACCGCTTTGGCCGCCGCCCAGTCCTGCTCGCTTCGCTACTTGGAGCCACTATCGACTACGCGATCATGGCGA
CCACACCCGTCCTGTGGATCCTCTACGCCGGACGCATCGTGGCCGGCATCACCGGCGCCACAGGTGCGGTTGCTGGC
GCCTATATCGCCGACATCACCGATGGGGAAGATCGGGCTCGCCACTTCGGGCTCATGAGCGCTTGTTTCGGCGTGGG
TATGGTGGCAGGCCCCGTGGCCGGGGGACTGTTGGGCGCCATCTCCTTGCATGCACCATTCCTTGCGGCGGCGGTGC
TCAACGGCCTCAACCTACTACTGGGCTGCTTCCTAATGCAGGAGTCGCATAAGGGAGAGCGTCGACCGATGCCCTTG
AGAGCCTTCAACCCAGTCAGCTCCTTCCGGTGGGCGCGGGGCATGACTATCGTCGCCGCACTTATGACTGTCTTCTT
TATCATGCAACTCGTAGGACAGGTGCCGGCAGCGCTCTGGGTCATTTTCGGCGAGGACCGCTTTCGCTGGAGCGCGA
CGATGATCGGCCTGTCGCTTGCGGTATTCGGAATCTTGCACGCCCTCGCTCAAGCCTTCGTCACTGGTCCCGCCACC
AAACGTTTCGGCGAGAAGCAGGCCATTATCGCCGGCATGGCGGCCGACGCGCTGGGCTACGTCTTGCTGGCGTTCGC
GACGCGAGGCTGGATGGCCTTCCCCATTATGATTCTTCTCGCTTCCGGCGGCATCGGGATGCCCGCGTTGCAGGCCA
TGCTGTCCAGGCAGGTAGATGACGACCATCAGGGACAGCTTCAAGGATCGCTCGCGGCTCTTACCAGCCTAACTTCG
ATCAtTGGACCGCTGATCGTCACGGCGATTTATGCCGCCTCGGCGAGCACATGGAACGGGTTGGCATGGATTGTAGG
CGCCGCCCTATACCTTGTCTGCCTCCCCGCGTTGCGTCGCGGTGCATGGAGCCGGGCCACCTCGACCTAATGGCCGA
GGAGCAGGACTGAtccgaaaaatggcgcacattgtgcgccatttttttttgGATCTGCGTTTCCTCAAACAGTTTAAA
TAAtgcagatccttagcgaaagctaaggattttttttg

>Pkan-bsdbsd-Pcat’-ATG-SKIKtag-FRT3-Cpf1s1 for dual Flp-POP cloning

aaagccacgttgtgtctcaaaatctctgatgttgcattgcacaagataaaaatatatcatcatgaacaataaaactg
tctgcttacataaacggaaaatttttttttcaaaagtacttgacaacataacggataacaattGtaatagattcaatt
gtaacgtaatacaaggaacgtgttATGGCAAAACCGCTGAGCCAAGAAGAAAGCACCCTGATTGAACGTGCAACCGC
AACCATTAATAGCATTCCGATTAGCGAAGATTATAGCGTTGCAAGCGCAGCACTGAGCAGTGATGGTCGcATTTTTA
CCGGTGTTAACGTGTATCATTTTACAGGTGGTCCGTGTGCAGAACTGGTTGTTCTGGGCACCGCtGCaGCAGCcGCA
GCtGGTAATCTGACCTGTATcGTTGCAATTGGTAATGAGAATCGTGGcATTCTGAGCCCGTGTGGTCGTTGTCGTCA
GGTTCTGCTGGATCTGCATCCGGGTATcAAAGCAATTGTGAAAGATTCAGATGGTCAGCCGACCGCAGTTGGaATTC
GTGAACTGCTGCCGAGCGGTTATGTTTGGGAAGGTTAAgttgtaaggattatgccATGGCCAAGCCTTTGTCTCAAG
AAGAATCCACCCTCATTGAAAGAGCAACGGCTACAATCAACAGCATCCCCATCTCTGAAGACTACAGCGTCGCCAGC
GCAGCTCTCTCTAGCGACGGCCGCATCTTtACaGGTGTCAATGTATAcCAcTTcACTGGGGGACCTTGTGCAGAACT
CGTGGTGCTGGGCACTGCgGCtGCgGCtGCAGCTGGCAACCTGACTTGTATCGTCGCGATCGGAAATGAGAACAGGG
GCATCTTGAGCCCCTGCGGACGGTGCCGACAGGTGCTTCTCGATCTGCATCCTGGGATCAAAGCCATAGTGAAGGAC
AGTGATGGACAGCCGACGGCAGTTGGGATTCGTGAATTGCTGCCCTCTGGTTATGTGTGGGAGGGCTAAtgcGCTGG
CAACTAGAAGGCACAGcgtaagaggttccaactttcaccatattgacataagatcactaccgggcgtataatttgag
ttatcgagattttcaggagctaaggaagctaaaATGtctaaaattaaatctggaagttcctattcttcaaatagtat
aggaacttcCAGGTCTTTCTCAAGCCGAcctttagagaagtcatttaataaggccact

>Cpf1s3-attPv-FRT3-spec(no start)-Ttrp-PEM7-zeo-Tlpp-TrrnC for dual Flp-POP cloning

gcggtttttttatgccagttgtttttgcttCTACTAATACAAAAGGCTCTAGACCAAAGGTCACTAATACTATCTAA
GTAGTTGATTCATAGTGACTGGATATGTTGCGTTTTGTCGCATTATGTAGTCTATCATTTAACCACAGATTAGTGTA
ATGCGATGATTTTTAAGTGATTAATGTTATTTTGTCATCCTTTtacaatgTAAGTTGTATATTTAAAATCTCTTTAA
TTATCAGTAAATTAATGTAAGTAGGTCATTATTAGTCAAAATAAAATCATTTGcgctgcttttgcacgtgaggaagt
tcctattcttcaaatagtataggaacttctCGcGAAGCGGTtATCGCCGAAGTATCGACTCAACTATCAGAGGTAGT
TGGCGTCATCGAGCGCCATCTCGAACCGACGTTGCTGGCCGTACATTTGTACGGCTCCGCAGTGGATGGCGGCCTGA
AGCCACACAGTGATATTGATTTGCTGGTTACGGTGACCGTAAGGCTTGATGAAACAACGCGGCGAGCTTTGATCAAC

GACCTTTTGGAAACTTCGGCTTCCCCTGGAGAGAGCGAGATTCTCCGCGCTGTAGAAGTCACCATTGTTGTGCACGA
CGACATCATTCCGTGGCGTTATCCAGCTAAGCGCGAACTGCAATTTGGAGAATGGCAGCGCAATGACATTCTTGCAG
GTATCTTCGAGCCAGCCACGATCGACATTGATCTGGCTATCTTGCTGACAAAAGCAAGAGAACATAGCGTTGCCTTG
GTAGGTCCAGCGGCGGAGGAACTCTTTGATCCGGTTCCTGAACAGGATCTATTTGAGGCGCTAAATGAAACCTTAAC
GCTATGGAACTCGCCGCCCGACTGGGCTGGCGATGAGCGAAATGTAGTGCTTACGTTGTCCCGCATTTGGTACAGCG
CAGTAACCGGCAAAATCGCGCCGAAGGATGTCGCTGCCGACTGGGCAATGGAGCGCCTGCCGGCCCAGTATCAGCCC
GTCATACTTGAAGCTAGACAGGCTTATCTTGGACAAGAAGAAGATCGCTTGGCCTCGCGCGCAGATCAGTTGGAAGA
ATTTGTCCACTACGTGAAAGGCGAGATCACCAAGGTAGTCGGCAAATAAtcccacagccgccagttccgctggcggc
attttgACTGCCATGGAAAATCGATGTTCTTCGGATCTGATCAGCACGTGTTGACAATTAATCATCGGCATAGTATA
TCGGCATAGTATAATACGACAAGGTGAGGAACTAAACCATGGCCAAGTTGACCAGTGCCGTTCCGGTGCTCACCGCG
CGCGACGTCGCCGGAGCGGTCGAGTTCTGGACCGACCGGCTCGGGTTCTCCCGGGACTTCGTGGAGGACGACTTCGC
CGGTGTGGTCCGGGACGACGTGACCCTGTTCATCAGCGCGGTCCAGGACCAGGTGGTGCCGGACAACACCCTGGCCT
GGGTGTGGGTGCGCGGCCTGGACGAGCTGTACGCCGAGTGGTCGGAGGTCGTGTCCACGAACTTCCGGGACGCCTCC
GGGCCGGCCATGACCGAGATCGGCGAGCAGCCGTGGGGGCGGGAGTTCGCCCTGCGCGACCCGGCCGGCAACTGCGT
GCACTTCGTGGCCGAGGAGCAGGACTGAtccgaaaaatggcgcacattgtgcgccatttttttttgGATCTGCGTTTC
CTCAAACAGTTTAAATAAtgcagatccttagcgaaagctaaggattttttttg

Table S2. The sequences of the homology arms used in the λ Red recombination experiments.

@phoU-trmE for oriT-POP cloning
Fwd: GCATGGTCATATTTTTATCAATAGCGCATTGCTATTTTCTCTGCACGCAA
Rev: TTACGTAATTTATAATCTTTAAAAAAAGCATTTAATATTGCTCCCC

@polB-leuD for oriT-POP cloning
Fwd: TAGCCTGGTTTCGTTTGATTGGCTGTGGTTTTATACAGTCATTGA
Rev:
ACGACGCCATTGCCGCTTATGAAGCAAAACAACCTGCGTTTATGAATTAATCCCCTTGCCCGGTCAAATGACCGGGC
TTTCCGCTATCGTCCACGTCATC

@after maoSP for oriT-POP cloning
Fwd: TAATGTCAGACTGATCAATATCAGCAATGGTTCGTGCAACTTTCAATAAC
Rev: AAGGAACACTGATCCATCTGGGGTTATCGATTCGTGCCTGGCAGCG

@lysS-yqeF for oriT-POP cloning
Fwd: CATTACGTTATGCTCACAACCCCGGCAAATGTCGGGGTTTTTTTA
Rev: TTTATGGTAAATTGCCCTCCATTCTGTTTAATTTGTAGATGATACGTTC

@polB-leuD for dual Flp-POP cloning
Fwd: GTATAGCCTGGTTTCGTTTGATTGGCTGTGGTTTTATACAGTCATT
Rev: CTTGCCCGGTCAAATGACCGGGCTTTCCGCTATCGTCCACGTCATC

@ygbL-gltA for dual Flp-POP cloning
Fwd: CTACGCTCTGCATTTGCCGAAAAGGGGATTGTTGTCGCAGCATAA
Rev: TCTGGCGTAGCTATACCCCTATTCTATATCCTTAAAGGACTCTGTT

@murJ-rne for dual Flp-POP cloning
Fwd: TTAAAACATATCATGAAACTGGGTATGTTTTGTCTGCCTGCTC
Rev: AAATTCATCGAATGGCATCCTTGCTAACCAACAATGCAAAATAGGC

@lysS-yqeF for dual Flp-POP cloning
Fwd: GTTCCCGGCGATGCGTCCGGTAAAATAAGCATTACGTTATGCTCAC
Rev: ATGGTAAATTGCCCTCCATTCTGTTTAATTTGTAGATGATACGTTC

@pgsA-yedP for dual Flp-POP cloning
Fwd: TCGCCGCATTGGTGCGCGCGAGTTTCAGATAACGCGCCCTATCGCCG
Rev: TCCCGACCCCCTCAGGGTCGGGATTTTTTTATTGTGCATTC

@glpC-menF for dual Flp-POP cloning
Fwd: TCCGTGCCCAATGTATGCGTTGCAACGCAGTGAAAATTCCTCTGA
Rev: AGTCGCATCATTACCGATTCATATCAATAATCTATTTTTGTAGCT

@icd-minE for AvrII site elimination
Fwd: GCGAGCGCGGCCCCTTCACATACATCTTTAGTACTGAGACTGTTTAA
Rev: GAAAACTGCGCCGAAGCGCAGTTTAATCGTTGATGGTACGACAATAATTC

A

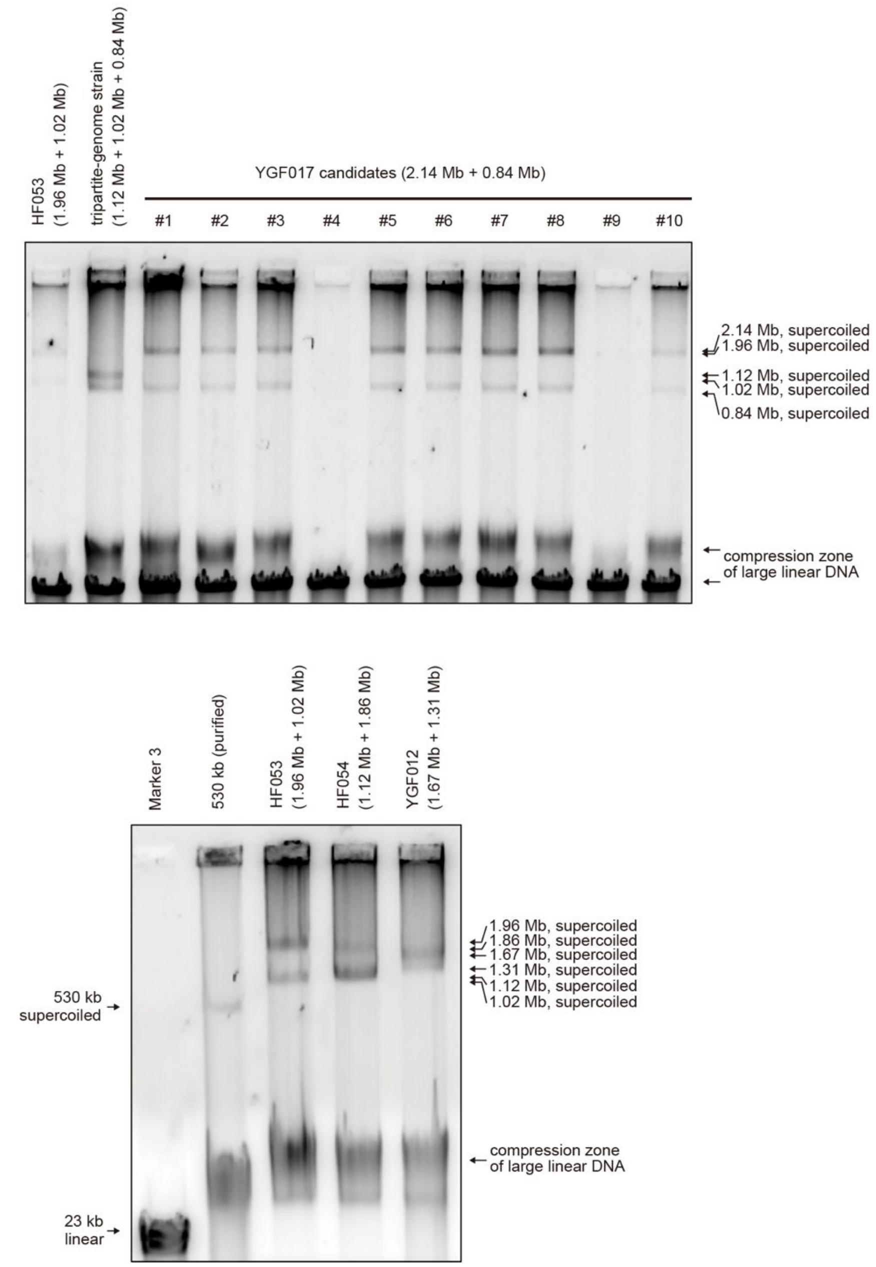

Figure S1. Developing bipartite-genome strains via dual Flp-POP cloning. (A) Agarose gel electrophoresis analysis (0.3% SeaKem Gold gel, 0.5x TBE, 40 V 120 min) of the SCR products from ten YGF017 candidate clones (chloramphenicol and spectinomycin resistant) was performed to check the bipartite-genome configuration. (B) Agarose gel electrophoresis analysis (0.3% SeaKem Gold gel, 0.5x TBE, 40 V 120 min) of the SCR products of bacterial chromosomes ranging from 1.02 Mb to 1.96 Mb in size.

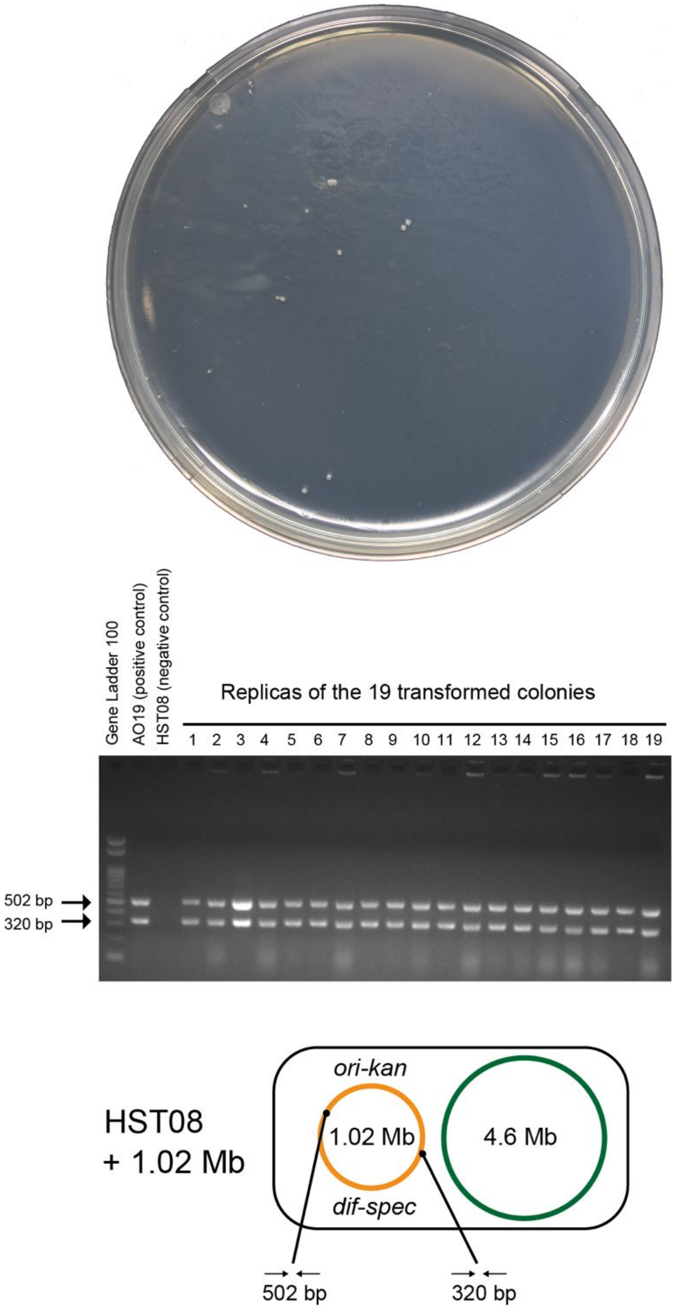

Figure S2. Electroporation of HST08 with a 1.02 Mb chromosome prepared by L-SCR. The photo of transformed colonies on an LB agar plate containing kanamycin and spectinomycin and the result of their colony PCR check with two sets of primers to detect two junctions specific to the 1.02 Mb chromosome. The primer pairs are (GAGACTGTAGAGCGTATGTAATCG & GTTGAATCAATGCTGCCAAGC) for 502 bp and (GTACTTCGCTGCACTGGC & GCGTCCGCAACCTGTTGAG) for 320 bp.

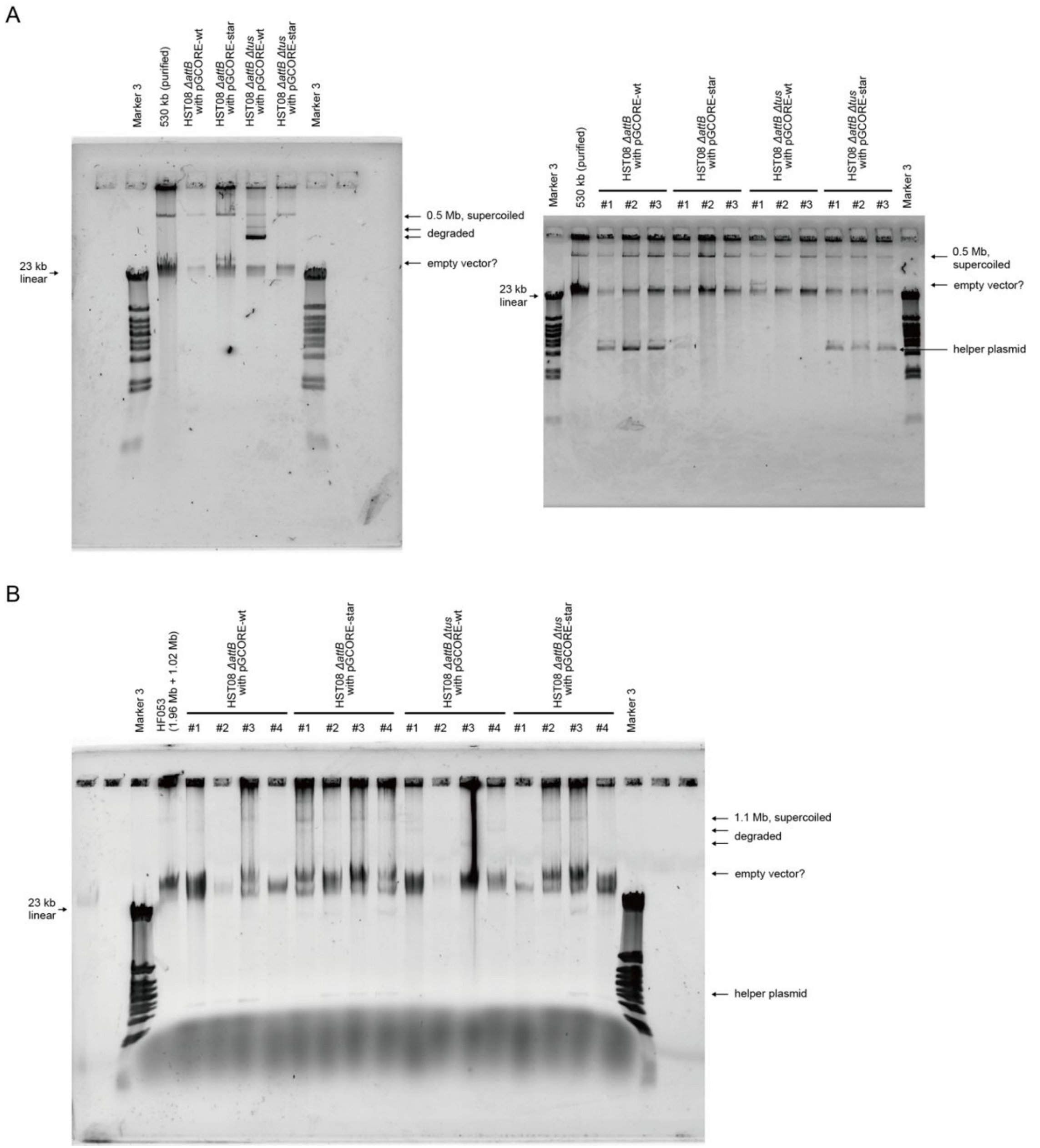

Figure S3. Assembling a portable 1.12 Mb chromosome. (A) Agarose gel electrophoresis analysis (0.5% High Quality Agarose (Recenttec K.K.), 0.5x TBE, 50 V 30 min) of the NucleoBond PC 20 column-purified chromosomes was performed to confirm the cloning of the left region (left panel) or the right region (right panel) of the 1.12 Mb region via the *oriT*-POP method into the pGCORE-star or pGCORE-wt BAC vector in HST08 Δ*attB* or HST08 Δ*attB* Δ*tus* carrying pKD46-int. (B) Agarose gel electrophoresis analysis (0.3% High Quality Agarose, 0.5x TBE, 40 V 90 min) of the SCR products from each four colonies (kanamycin, Zeocin, and spectinomycin resistant) under the four conditions. Several colonies selected on the second *oriT*-POP cloning step carry a large (probably 1.12 Mb) chromosome. Some colonies may also carry a degraded chromosome and an empty BAC vector as well as the helper plasmid. By an unknown reason, the helper plasmid was hardly curable after cloning the right region.

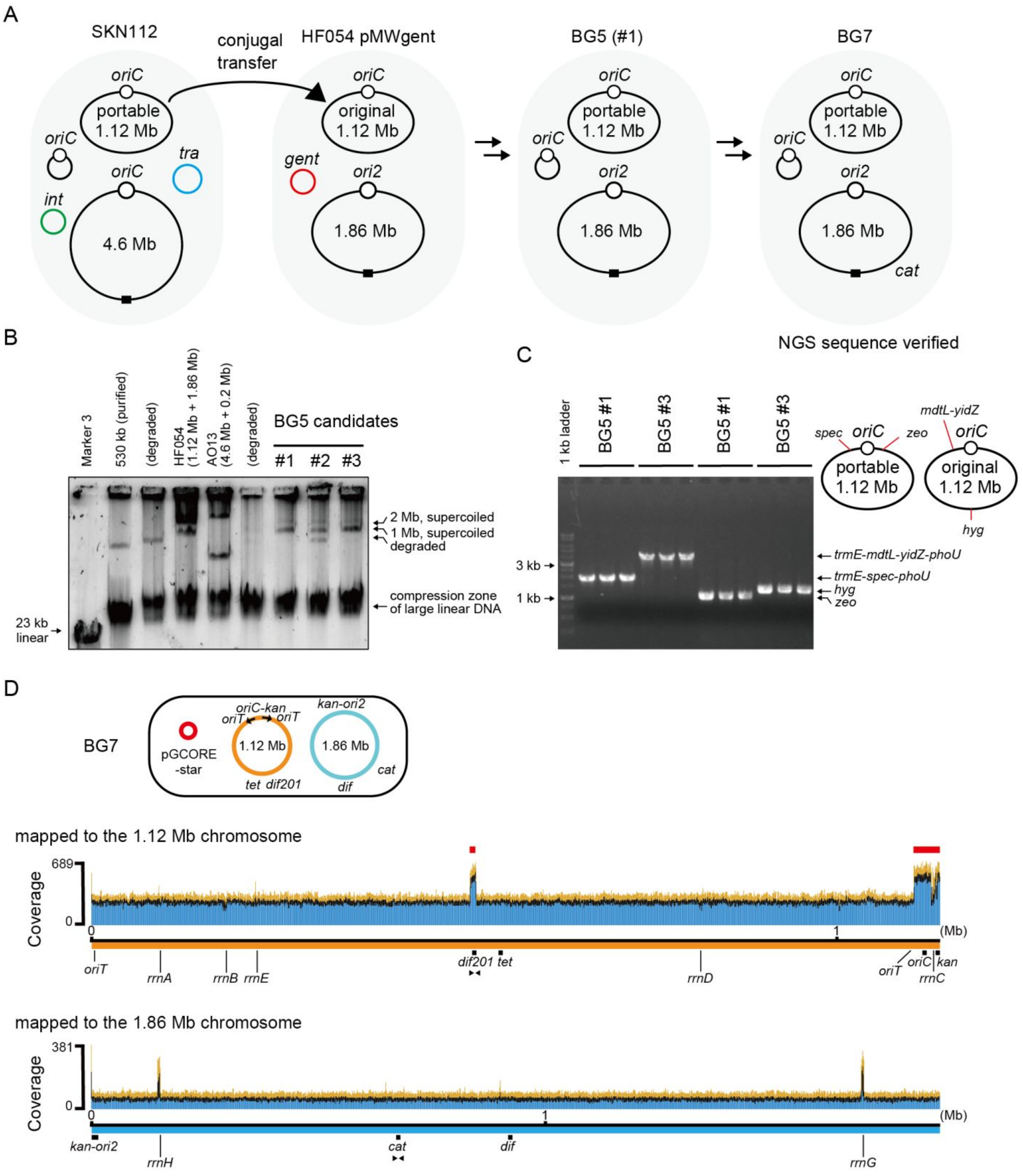
A
SKN112
conjugal transfer
HF054 pMWgent
BG5 (#1)
BG7
oriC
portable 1.12 Mb
original 1.12 Mb
tra
int
gent
4.6 Mb
ori2
1.86 Mb
cat
NGS sequence verified
B
Marker 3
530 kb (purified)
(degraded)
HF054 (1.12 Mb + 1.86 Mb)
AO13 (4.6 Mb + 0.2 Mb)
(degraded)
BG5 candidates
#1
#2
#3
2 Mb, supercoiled
1 Mb, supercoiled
degraded
compression zone of large linear DNA
23 kb linear
C
1 kb ladder
BG5 #1
BG5 #3
BG5 #1
BG5 #3
3 kb
1 kb
trmE-mdtL-yidZ-phoU
trmE-spec-phoU
hyg
zeo
spec
zeo
mdtL-yidZ
oriC
portable 1.12 Mb
original 1.12 Mb
hyg
D
BG7
pGCORE -star
oriC-kan
oriT
oriT
1.12 Mb
tet
dif201
kan-ori2
1.86 Mb
cat
dif
mapped to the 1.12 Mb chromosome
Coverage
689
0
0
1
(Mb)
oriT
rrnA
rrnB
rrnE
dif201
tet
rrnD
oriT
oriC
rrnC
kan
mapped to the 1.86 Mb chromosome
Coverage
381
0
0
1
(Mb)
kan-ori2
rrnH
cat
dif
rrnG

E

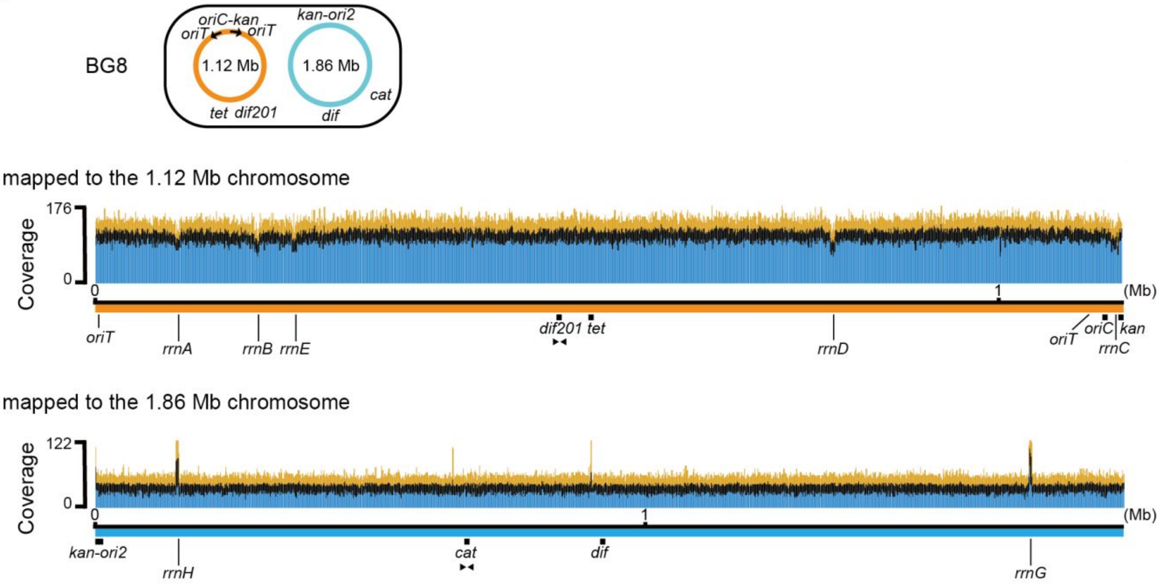

Figure S4. Portable chromosome for partial genome swap. (A) Procedure for the conjugal transfer of the portable 1.12 Mb chromosome to replace the original 1.12 Mb chromosome in HF054 carrying an empty plasmid with a gentamicin resistance gene (*gent*). "*tra*" and "*int*" denote the RP4 *tra* gene clusters and the HK022 integrase gene, respectively, cloned in helper plasmids. Strain BG5 and its chloramphenicol-resistant derivative BG7 should have the depicted bipartite-genome configuration. By an unknown reason, an empty pGCORE-star BAC vector has accompanied with the 1.12 Mb chromosome during the cell engineering. (B) Agarose gel electrophoresis analysis of the SCR products from three BG5 candidate clones (spectinomycin and gentamicin resistant). It was revealed that clones No. 1 and 3 have two chromosomes, while the clone No. 2 may have an extra degraded chromosome. (C) Colony-direct PCR analyses of the BG5 candidate clones No. 1 and 3 was performed and showed that the clone No. 1 has the portable 1.12 Mb chromosome, while the clone No. 3 retains the original 1.12 Mb chromosome. (D, E) NGS analysis of the genomes of BG7 (D) and its plasmid-free derivative BG8 (E). The *oriC, ori2, oriT, kan, cat, tet, dif/dif201* loci, the seven *rrn* operons, and the colony PCR sites (indicated with arrows) are shown on the maps.

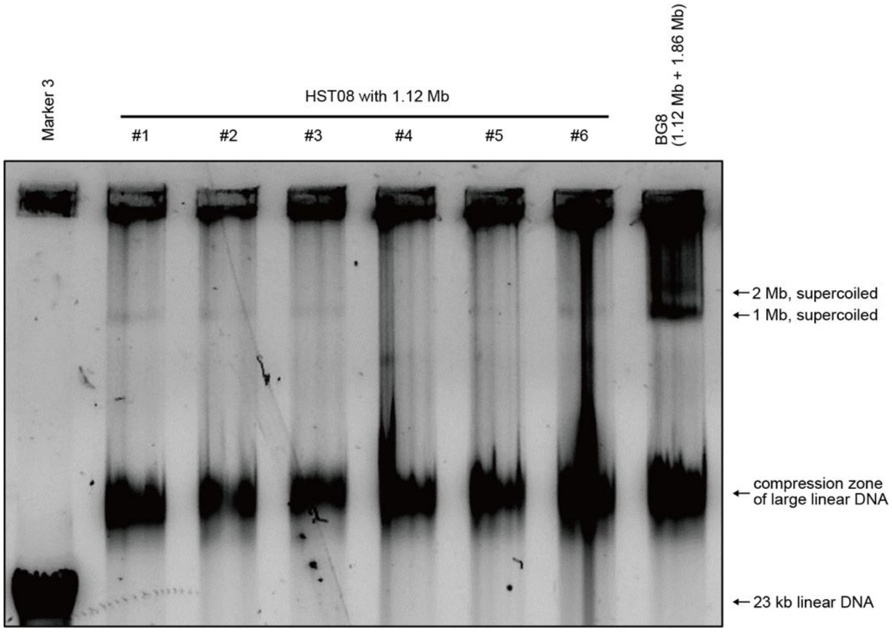

Figure S5. Detecting the portable 1.12 Mb chromosome. L-SCR products of the 1.12 Mb chromosomes were introduced into *E. coli* HST08 cells via electroporation. Agarose gel electrophoresis analysis (0.3% SeaKem Gold gel, 0.5x TBE, 40 V 90 min) of the L-SCR products from BG8 and the transformed HST08 clones was performed to detect the intact 1.12 Mb chromosome in the transformed HST08 clones.

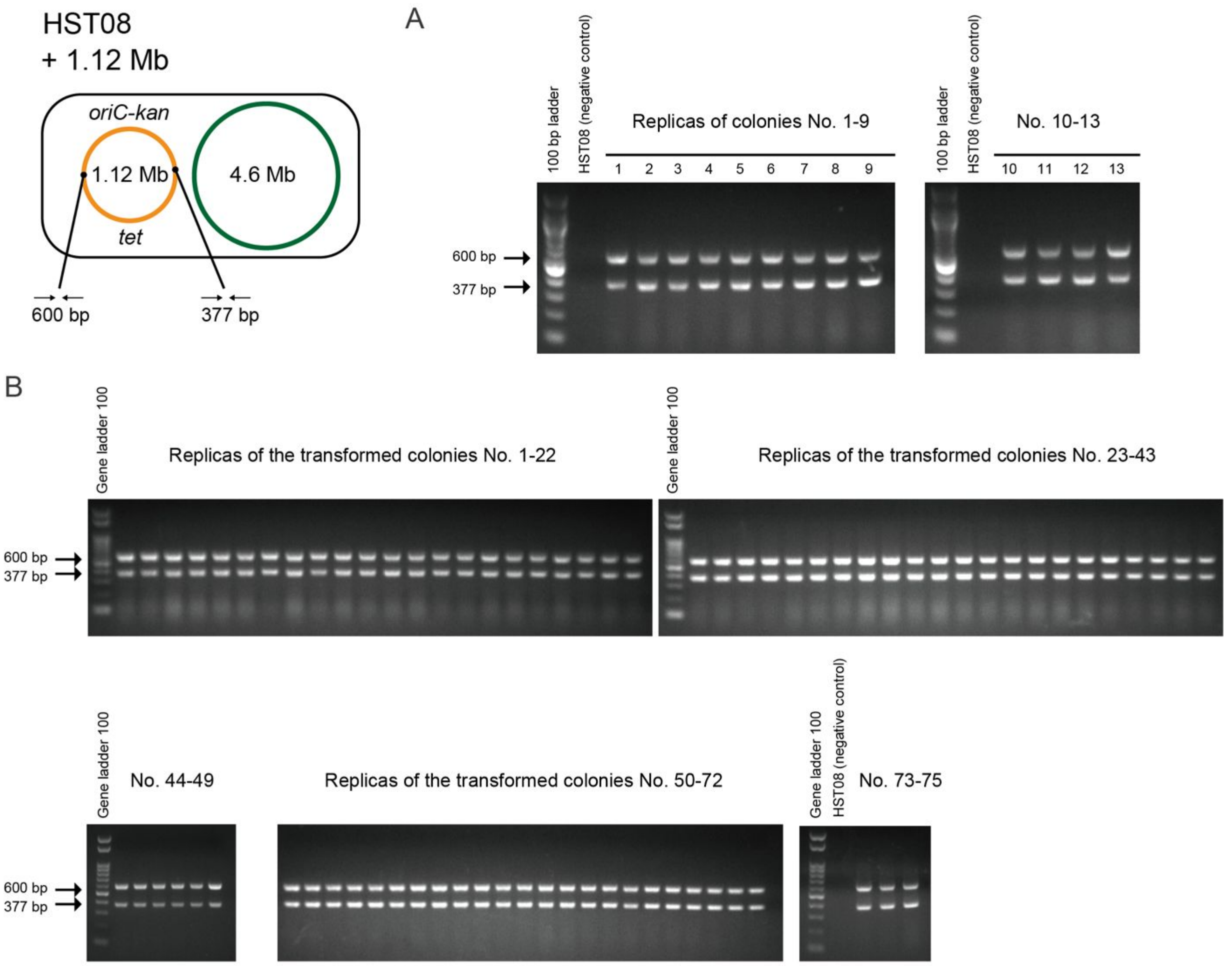

Figure S6. Electroporation of HST08 with a 1.12 Mb chromosome prepared by L-SCR. Two sets of PCR primers were used to detect two junctions specific to the 1.12 Mb chromosome. The primer pairs are (GCGTTGTTGCTAAAGTTCTGG & GATGATCAAACGCTTTCAGGC) for 600 bp and (CCAATTATCCTAAAACGCCATCG & AAGGCATTGTATCGCAGAAAAGTG) for 377 bp. (A, B) Colony direct PCR analyses of the replicas of colonies appeared on selection LB agar plates containing kanamycin (30 µg/ml) and tetracycline (10 µg/ml) after electroporation using 10 vials of competent cells (A) and 18 vials of competent cells (B). Colonies appeared after 2 days incubation (colony No. 10-13 for A and colony No. 50-75 for B) were also analysed by PCR.

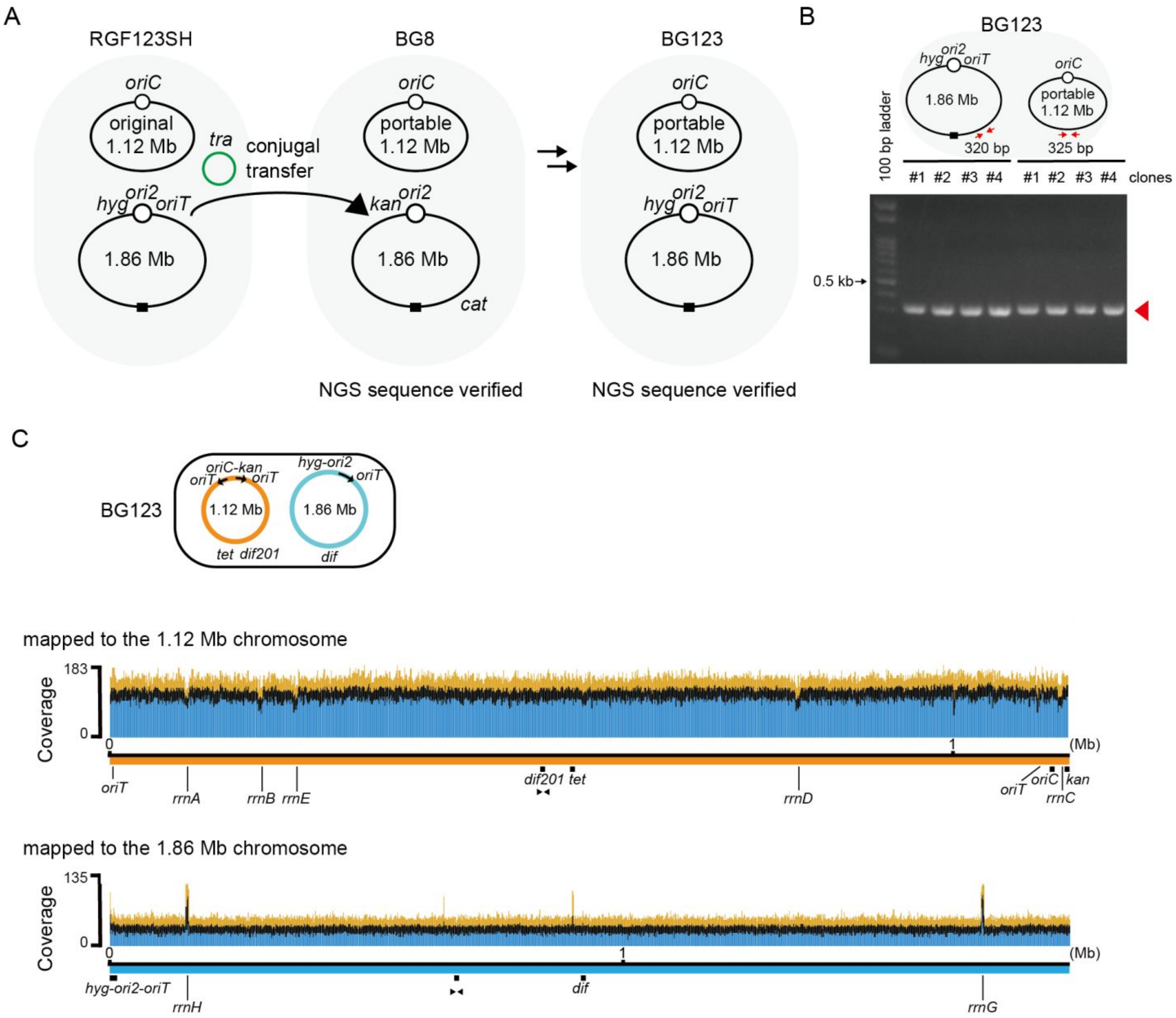

Figure S7. Two step genome swap. (A) Procedure for the conjugal transfer of the 1.86 Mb chromosome of RGF123SH to BG8, a plasmid-free derivative of BG7, to develop BG123. RGF123SH had been developed form RGF123 by replacing the *oriC-kan* locus of the 1.86 Mb chromosome with a *hyg-spec* cassette via λ-red recombination. The whole genome sequences of BG8 and BG123 strains were verified by NGS. (B) Colony-direct PCR analyses of four BG123 candidate clones were performed to confirm the depicted chromosome configuration. A few other loci were also checked by PCR (data not shown). (C) NGS analysis of BG123. The *oriC, ori2, oriT, kan, cat, hyg, tet, dif/dif201* loci, the seven *rrn* operons, and the colony PCR sites (indicated with arrows) are shown on the maps.
